# HIF1A-AS2 promotes the metabolic reprogramming and progression of colorectal cancer via miR-141-3p/FOXC1 axis

**DOI:** 10.1038/s41419-024-06958-2

**Published:** 2024-09-03

**Authors:** Xinyang Zhong, Yaxian Wang, Xuefeng He, Xinxin He, Zijuan Hu, Huixia Huang, Jiayu Chen, Keji Chen, Ping Wei, Senlin Zhao, Yilin Wang, Hong Zhang, Bo Feng, Dawei Li

**Affiliations:** 1 Department of Colorectal Surgery, Fudan University Shanghai Cancer Center, Shanghai, China; 2 Department of Oncology, Shanghai Medical College Fudan University, Shanghai, China; 3 Cancer Institute, ZJU-UCLA Joint Center for Medical Education and Research, The Second Affiliated Hospital, Zhejiang University School of Medicine, Hangzhou, China; 4 Department of Gastrointestinal Surgery, Guangxi Medical University Cancer Hospital, Nanning, China; 5 Department of Pathology, Fudan University Shanghai Cancer Center, Shanghai, China; 6 Cancer Institute, Fudan University Shanghai Cancer Center, Shanghai, China; 7 Institute of Pathology, Fudan University, Shanghai, China; 8 Department of Hepatic Surgery, Fudan University Shanghai Cancer Center, Shanghai, China; 9 Colorectal Tumor Surgery Ward, Department of General Surgery, Shengjing Hospital of China Medical University, Shenyang, China; 10 Department of General Surgery, Ruijin Hospital, Shanghai Jiao Tong University School of Medicine, Shanghai, China

**Keywords:** Colorectal cancer, Cell migration

## Abstract

lncRNA can regulate tumorigenesis development and distant metastasis of colorectal cancer (CRC). However, the detailed molecular mechanisms are still largely unknown. Using RNA-sequencing data, RT-qPCR, and FISH assay, we found that HIF1A-AS2 was upregulated in CRC tissues and associated with poor prognosis. Functional experiments were performed to determine the roles of HIF1A-AS2 in tumor progression and we found that HIF1A-AS2 can promote the proliferation, metastasis, and aerobic glycolysis of CRC cells. Mechanistically, HIF1A-AS2 can promote FOXC1 expression by sponging miR-141-3p. SP1 can transcriptionally activate HIF1A-AS2. Further, HIF1A-AS2 can be packaged into exosomes and promote the malignant phenotype of recipient tumor cells. Taken together, we discovered that SP1-induced HIF1A-AS2 can promote the metabolic reprogramming and progression of CRC via miR-141-3p/FOXC1 axis. HIF1A-AS2 is a promising diagnostic marker and treatment target in CRC.

## Introduction

In 2022, colorectal cancer (CRC) remains the second leading cause of cancer-related deaths and the third most commonly diagnosed tumor, imposing a significant economic and social burden on many countries [1]. Despite various treatment options, such as surgery resection, chemotherapy, targeted therapy, and immune therapy, a considerable number of patients experience disease progression. Thus, investigating the pathogenesis and discovering effective therapeutic targets in CRC is critical.

Metabolic reprogramming is a hallmark of cancer [2]. One of the most widely recognized metabolic characteristics of carcinoma cells is the Warburg effect, which refers to the capability to metabolize substantial amounts of glucose to produce lactate, regardless of the availability of oxygen, hence also termed aerobic glycolysis [3, 4]. Aerobic glycolysis can alter the extracellular tumor microenvironment (TME), satisfy the energetic and biosynthetic needs of cancer cells, and maintain the redox homeostasis of tumor cells [5]. Tumor cells absorb significant amounts of glucose and release lactate, which promotes tumor growth and leads to a heterogeneous TME characterized by extracellular acidosis, hypoxia, and nutrition deprivation [3]. On the one hand, the abnormal TME can impair the function of immune cells; on the other hand, it can activate the transcriptional factors or the signaling pathways in CRC in an abnormal manner [6, 7]. We summarized the oncogenic roles of the Warburg effect in CRC and suggested that targeting this metabolic feature has great potential in CRC therapy [8].

Recently, the emerging functions of long non-coding RNAs (lncRNAs) in tumor biology have got much attention. Besides their ability to regulate transcription and chromatin/histone remodeling, lncRNAs act as miRNA sponges and protein scaffolds, modify mRNA and protein stability, and play a significant role in cancer metabolism [9, 10]. Notably, our previous research suggested that lncRNA MIR17HG is abnormally upregulated in CRC and promotes colorectal cancer liver metastasis (CRLM) via upregulating the expression of HK1, a key enzyme in glycolysis [7]. Moreover, lncRNAs can be packaged into exosomes and exported into the tumor microenvironment to influence the biological behavior of nearby cells [11]. Additionally, lncRNAs can be applied to evaluate the prognosis of patients or the immune microenvironment of CRC [12, 13]. Hence, exploring the detailed mechanisms by which lncRNAs regulate tumor development is all-important.

LncRNA HIF1A-AS2 has been reported to promote tumor progression and is found to be abnormally expressed in some cancers [14]. However, little is known regarding the oncogenic function of HIF1A-AS2 in CRC. Here, transcriptomic data analysis was employed, and we discovered a significantly higher level of HIF1A-AS2 in CRLM tissues than in primary CRC. Further research revealed that HIF1A-AS2 expression is associated with prognosis in CRC patients. Transcriptional factor SP1 can promote HIF1A-AS2 transcription, and HIF1A-AS2 then promotes the malignant phenotypes, including metastasis and glycolysis of CRC. Mechanistically, HIF1A-AS2 exerts its oncogenic functions by sponging miR-141-3p, thereby upregulating FOXC1 expression. Moreover, we found that HIF1A-AS2 can be packaged into exosomes, enhancing the malignancy of nearby tumor cells.

## Methods and materials

### Cell culture and transfection

Human embryonic kidney cell line (HEK-293T), CRC cell lines (HCT15, HCT8, DLD1, RKO, SW480, HCT116, SW620, LoVo, HT29, Caco2, COLO205, and SW1116), and normal colon epithelial cell line (NCM460) were purchased from the Cell Bank of the Chinese Academy of Sciences (Shanghai, China). RPMI-1640 medium (Gibco, USA) was used to culture HCT15, HCT8, and DLD1 cells, while DMEM medium (Gibco, USA) was applied to cultivate HCT116, RKO, SW480, SW620, LoVo, HT29, Caco2, COLO205, SW1116, NCM460, and HEK-293T. Both media contain 10% fetal bovine serum (FBS) and 1% penicillin/streptomycin. PCR method was used to routinely check for mycoplasma contamination.

To knock down HIF1A-AS2 in CRC cells, short hairpin RNAs (shRNAs) were cloned into the pGLV3/H1/GFP/puro vector (sh-HIF1A-AS2#1/sh1 and sh-HIF1A-AS2#2/sh2) (Shanghai Fanxu Technology). Full-length HIF1A-AS2 was cloned into pCDH-CMV-MCS-EF1-copGFP-T2A-Puro plasmid (ex-HIF1A-AS2/HIF1A-AS2) to overexpress HIF1A-AS2. HEK-293T cells were transfected with the knockdown/overexpressed plasmids, psPAX2, and pMD2.G, using a transfection reagent. After 48 h, the culture medium of HEK-293T cells (containing lentivirus particles encapsulating knockdown/overexpression constructs) was harvested, filtered using a percolator whose bore diameter is 0.45 μm (Millipore, USA), and applied to transfect CRC cells to knock down or overexpress HIF1A-AS2. Stable cell lines were selected using a culture medium containing 5 µg/mL puromycin. Short hairpin RNAs against FOXC1 (sh-FOXC1) and relevant overexpression plasmids (ex-FOXC1), were also designed. SP1, FOXP3, E2F1, wild-type and mutant HIF1A-AS2 were cloned into pcDNA3.1 vector. siRNAs against FOXP3, SP1, E2F1, and the mimics or inhibitors of miR-141-3p were transfected into CRC cell line to change the expression of relevant genes. Supplementary Table 1 summarized the sequences of shRNAs and siRNAs, and Supplementary Table 2 summarized the sequences of the mimics and inhibitors.

### Patients and tissue samples

To perform RNA sequencing, we used primary CRC tissues and corresponding CRLM samples from eight patients who did not receive any neoadjuvant treatment before surgery. We also extracted tissue RNA from tumor and adjacent normal tissues of 106 CRC patients who underwent surgery at Fudan University Shanghai Cancer Center (FUSCC) between 2014 and 2017 (FUSCC cohort). Among these 106 tumor samples, we extracted miRNA from 74 of them. We recorded the SUV max values from 18F-FDG PET/CT examination for 30 out of the 106 patients who have relevant data to explore its relationship with HIF1A-AS2 expression. All the PET/CT photos were obtained with informed consent. Supplementary Table 3 summarizes the clinical information of these patients. Furthermore, we used tissue microarrays (TMA), consisting of 272 CRC patients (TMA cohort), to measure the RNA level of HIF1A-AS2 and discover its correlation with SP1. Supplementary Table 4 summarizes the clinical stages of the patients in the TMA cohort. Plasma-derived exosomes were extracted, as explained below, to compare the exosomal expression of HIF1A-AS2 between normal people and CRC patients. Our study was approved by the Ethics Committee on Scientific Research of Fudan University Shanghai Cancer Center, and all the individuals involved in this study provided written informed consent for the use of clinical samples in medical research. All methods were performed in accordance with the relevant guidelines and regulations.

### RNA extraction and real‑time quantitative polymerase chain reaction (RT‒qPCR)

TRIzol reagent (Invitrogen, USA) was applied to extract RNA, which was reversely transcribed into cDNA by reverse transcriptase (ABclonal, China). miRNA was extracted using miRNA Isolation Kit R6842 (Omega Bio-Tek, China), and first-strand cDNA was synthesized using miRNA First Strand cDNA Synthesis kit (Sangon Biotech, China). PARIS Kit (Invitrogen, USA) was applied to find out the subcellular localization of HIF1A-AS2. RT‒qPCR analysis was performed to detect the expression of relevant genes using the SYBR Green PCR Kit (Taraka, Japan) and ABI 7900 real-time PCR Detection System (Applied Biosystems, USA). β-actin and U6 served as the internal reference. All the experiments were performed in triplicate, and primers are shown in Supplementary Table 5.

### Immunohistochemical (IHC) staining and fluorescence in situ hybridization (FISH) assay

Tumor tissues embedded in paraffin were cut into slices. The paraffin section was deparaffinized using xylene and rehydrated with ethanol solutions. Then, slides were heated in citrate buffer and blocked with 1% BSA. Next, tissues were incubated with diluted primary antibodies at 4° overnight. The list of primary antibodies is included in Supplementary Table 6. After washing out the primary antibody, the slides were incubated with biotinylated secondary antibodies and streptavidin-conjugated horseradish peroxidase (HRP) for 30–45 min. The HRP signal was detected using a DAB chromogenic kit (Gene Tech, China). Hematoxylin was applied to stain the nucleus, and hydrochloric acid in ethanol was used to differentiate tissues. The slides have four levels of color intensity (0 refers to no staining, 1 to weak, 2 to moderate, and 3 to strong) and five levels of the percentage of positively stained cells (0 refers to no positively stained cells, 1 to 1–20%, 2 to 21–50%, 3 to 51–80% and 4 to 81–100%). Two independent pathologists scored each slide by multiplying the above two scores, and the final IHC staining score of each sample ranges from 0 to 12.

Cy3-labeled HIF1A-AS2 probe, 18S, and U6 (RiboBio, China) were synthesized. The FISH assay was performed on tissue microarrays, and cell creeps using a FISH Kit (RiboBio, China). HIF1A-AS2 expression in the TMAs was evaluated by two independent pathologists. The light intensity of HIF1A-AS2 in TMAs was divided into three levels: 2 = high staining and 1 = low staining, while 0 = no staining. The percentage of HIF1A-AS2 positive cells was divided into four levels: 4 = 75~100%; 3 = 50~74%; 2 = 25~49% and 1 = 1~24%. Similarly, the FISH score of every sample was obtained by multiplying the two indicators above, ranging from 0 to 8.

### Cell proliferation, invasion, and metastasis assays

As for the CCK8 assay, 1000 treated CRC cells were seeded into a single well in 96-well plates. We then added CCK-8 assay solution (YEASEN, China) to each well and incubated the plate in a dark incubator for 2 h. We measured the absorbance of each well at 24-h intervals from day 1 to day 4. We also used a clone formation assay to evaluate proliferative activity. Briefly, 1.5 ml culture media containing 1000 treated CRC cells were added into each well. The incubation time of every independent experiment was equal. After about two weeks, cells were fixed in 4% paraformaldehyde (ServiceBio, China) for 30 min and stained with 1% crystal violet (YEASEN, China). The invasive ability of differently-treated cells was estimated by transwell invasion assay. Diluted Matrigel (BD Science, USA) was added to the upper chamber, and 600 μl complete medium was then added to the lower chamber. Then, 200 μl cell suspension containing 20,000 cells was added to every upper chamber, and cells were cultured for 48 h, after which they were fixed and stained. After scraping off the cells in the upper chamber, invaded cells were photographed and calculated. Additionally, we also assessed migration ability using the wound healing assay, which has been illustrated elsewhere [12]. Experiments were performed in triplicate.

### Western blot

To extract proteins from human CRC cells, we lysed them using RIPA buffer for 10 min on ice. Subsequently, we further lysed the mixture using ultrasonic waves. We quantified the protein concentration using a BCA reagent (Pierce™ BCA Protein Assay Kit, Thermo Fisher Scientific) and added 4× loading buffer and RIPA buffer according to the relevant concentration of each sample. We then denatured the protein using a 95° metal bath. We electrophoresed equal amounts of protein on SDS-PAGE gels and transferred them to a PVDF membrane (Millipore, Germany) with a 0.22 μm pore size. The voltage, current, and time of each experiment depended on the size of the target protein. The protein in the PVDF membrane was blocked using 5% milk, and then the membrane was incubated with primary antibodies for at least 10 h on the shaker at 4 °C. Next, the membrane was incubated with a secondary antibody for 1 h at room temperature. Finally, the exposure was carried out under the exposure machine. The internal reference was β-actin, and Supplementary Table 6 showed all the antibodies used in this research. The expression of the protein was quantified using Image J software.

### Glucose consumption, lactate production, and Seahorse assays

Glucose Uptake-Glo™ Assay (Promega) and Lactate-Glo™ Assay (Promega) were applied to test the glycolytic ability of CRC cells. Moreover, the extracellular acidification rate (ECAR) assay was performed to further measure the glycolytic level of differently-treated cells according to relevant protocols. On the first day, the sensors were submerged in the calibrant (Seahorse Bioscience, USA) overnight in a CO_2_-free incubator. Treated CRC cells were seeded into a 96-well plate (Seahorse Bioscience, USA) at a density of 20,000 cells per well and cultured for 24 h. On the next day, a fresh medium with two mM glutamine replaced the previously-added medium. Then, the 96-well plate was put in a CO_2_-free incubator for 60 min. Glucose, oligomycin, and 2-DG were added sequentially to each well. XF96 Extracellular Flux Analyzer (Seahorse Bioscience) was used to measure the ECAR of CRC cells. Experiments were performed in triplicate.

### RNA pull-down assay

RNA pull-down assay was performed as previously described to find out miRNAs that specifically bind to HIF1A-AS2 [15, 16]. In short, biotin-labeled probe targeting HIF1A-AS2 and control probe were incubated with M-280 streptavidin-coupled Dynabeads (Invitrogen, USA) for 2 h at room temperature. CRC cells were lysed and incubated with probe/beads complex overnight at 4 °C. Then, the probe/beads/RNA complex was washed for three times. The expression of miRNA and HIF1A-AS2 were determined using qRT-PCR.

### RNA immunoprecipitation (RIP) assay

RIP assay was done using the Magna RIP™ RNA-Binding Protein Immunoprecipitation Kit (Millipore, Billerica, MA, USA). In brief, 50 μl magnetic beads were combined with 5 μl anti-Ago2 antibody or immunoglobulin G. This mixture was incubated at room temperature for 30 min. Then, the antibody/beads complex was incubated with the split product of CRC cells overnight at 4 °C with rotation. On the second day, protein K (Millipore, USA) was added to digest the proteins in each immunoprecipitated, and RT-qPCR was applied to quantify the dissolved RNA. Experiments were performed in triplicate.

### Chromatin immunoprecipitation (ChIP) assay

ChIP assay was performed to explore the interaction between the HIF1A-AS2 promoter and potential transcription factors (TFs) that regulate HIF1A-AS2 transcription using The SimpleChIP Plus Sonication Chromatin IP Kit (CST, USA). Cells were fixed in 1% formaldehyde, and the cross-linking was stopped by adding glycine. Then, cells were lysed, and the DNA was digested using a micrococcal nuclease. Ultrasonic waves were used to shear the DNA to produce 150–900 bp fragments. Subsequently, the digested DNA fragments were incubated with anti-SP1/IgG antibody overnight at 4 °C with rotation. The next day, samples were washed and heated at 65 °C for 30 min while vortexed to elute chromatin from the beads. Finally, the chromatin DNA was purified and quantified using RT-qPCR. The primers used in ChIP-qPCR assays are listed in Supplementary Table 5. Experiments were performed in triplicate.

### Dual-luciferase reporter assay

The full-length HIF1A-AS2, 3’ untranslated region (UTR) of FOXC1, as well as the corresponding mutant versions of HIF1A-AS2 and mutant FOXC1 3′UTR (Shanghai Fanxu Technology, China), were cloned into the pmirGLO vector. miRNA mimics/negative controls were co-transfected with dual-luciferase reporter plasmids using lipo3000 (Invitrogen, USA). We measured the firefly and Renilla luciferase activities of CRC cells using a Dual-Luciferase Assay System (Promega) following the manufacturer’s protocol. The relative luciferase activity was calculated as the ratio of firefly/ Renilla luciferase activities. We inserted the wild-type and mutant fragments of the HIF1A-AS2 promoter into pGL3-basic plasmids to evaluate the transcriptional activity of promoter fragments. We also transfected an equal amount of pRL-TK plasmid into each group, which served as an internal control. Similarly, relative luciferase activities were determined, as mentioned above. All the experiments were performed in triplicate.

### Exosome extraction and exosome uptake assay

Exosomes derived from cell supernatant were extracted using ultracentrifuge method. We cultured HCT116 and DLD1 cells in exosome-free culture medium for 48 h. After 48 h, we collected the culture medium, which was centrifuged at 800 × *g* for 5 min to remove cells. The supernatant was further centrifuged at 2000 × *g* for 10 min to remove the cell debris. Subsequently, the supernatant was filtered through a membrane filter with a 0.22-μm-diameter pore size (Millipore, USA). The centrifuge tubes were further centrifuged at 100,000 × *g* for 110 min. After the first-round ultracentrifugation, we carefully removed the medium, and the pellet was washed using 20 ml PBS. Second-round centrifugation was performed, whose condition was the same as the first-round centrifugation. Finally, the PBS was removed, and exosomes were resuspended in ice-cold PBS. The concentration of exosomes was measured using BCA method (Pierce™ BCA Protein Assay Kit, Thermo Fisher Scientific). After measuring the protein concentration of each sample, we used PBS to dilute them to 20 μg/0.1 mL and store them at –80 °C.

Electron microscopy was used to visualize the morphology of the exosomes while nanoparticle tracking analysis (NTA) were used to quantify the size and concentration of the exosomes. The protein markers of exosomes were identified by western blot. Antibodies against TSG101 (1:1000), HSP70 (1:1000), and Annexin A1 (1:1000) were used in this analysis. Exosome uptake assay was performed using Exosome Staining Quick Kit (H.Wayen technology, China) by manufacturer’s protocol. The uptake of exosomes was visualized with a fluorescence microscope (Olympus, Japan).

Exosomes from the plasma of healthy subjects and CRC patients were extracted using an Exosome purification kit (Abclonal, China) following the relevant instructions. Exosomal HIF1A-AS2 level in healthy subjects and CRC patients was measured using RT-qPCR.

### Mouse model

We used male BALB/c nude mice (4-week-old) from Vital River Laboratories, China, to establish the subcutaneous tumor model and colorectal cancer liver metastasis model. Briefly, we injected 4 × 10^6^ stably-knockdown DLD1 cells or their negative control suspended with 100 μl PBS subcutaneously into the right flank of each mouse (*n* = 5 mice/group). Similarly, we injected 4 × 10^6^ stably-overexpressed HCT15 cells or their negative control to find out whether HIF1A-AS2 overexpression could promote tumor growth (*n* = 5 mice/group). Tumor size was measured every four days. Tumor volume was calculated using the formula: 0.50 × length × width^2^. To explore whether exosomal HIF1A-AS2 can influence tumor proliferation, we first injected 4 × 10^6^ HCT116 cells subcutaneously in each mouse (*n* = 10) and then obtained exosomes derived from HCT116 cells that stably-overexpressed HIF1A-AS2 or HCT116 cells that normally expressed HIF1A-AS2. On the fourth day, we injected 100 μl of PBS containing exosomes (20 μg/0.1 mL) that overexpressed HIF1A-AS2 or normally expressed HIF1A-AS2 into the tail vein (*n* = 5 mice/group) every three days. On the twentieth day, we excised, weighed, photographed, and fixed the tumors.

To establish a CRLM model and evaluate the role of HIF1A-AS2 in metastasis, we injected 1 × 10^6^ DLD1 cells or 2 × 10^6^ HCT15 cells (suspended in 100 μl PBS) into the spleens of mice (*n* = 9 mice/group). Mice were divided into two groups: one group (*n* = 4) was sacrificed after 5 weeks (DLD1 cells) or 6 weeks (HCT15 cells) to obtain the liver specimens. One group (*n* = 5) was used to monitor the survival time. To evaluate the effect of exosomal HIF1A-AS2 on tumor metastasis, we injected 1 × 10^6^ HCT116 cells into the spleen of mice (*n* = 9) and then injected 100 μl PBS containing 20 μg exosomes that overexpress/normally express HIF1A-AS2 through the tail vein every five days from the fifth day. Similarly, mice were divided into two groups: one group (*n* = 4) was sacrificed after 5 weeks to obtain the liver specimens. One other group (*n* = 5) was used to monitor the survival time. All the mice were performed luminescence experiment at day 35. The experimenter was not blinded to the assignment of the groups and the evaluation of the results. All animal studies were approved by the Committee on the Ethics of Animal Experiments of Fudan University. All methods were performed in accordance with the relevant guidelines and regulations.

### Bioinformatics analysis

Several online datasets, including GSE147602, GSE159216, GSE48267, GSE41665, and TCGA, were analyzed. Differentially-expressed lncRNAs (DELs) between eight primary CRC tissues and relevant CRLM samples and differentially-expressed genes between the NC group and HIF1A-AS2 knockdown group were analyzed using the limma R package. We performed the Kyoto Encyclopedia of Genes and Genomes (KEGG) analysis and gene set enrichment analysis (GSEA) analysis to explore the potential mechanisms through which HIF1A-AS2 regulates tumor progression. Gene set “h.all.v7.4.entrez.gmt” was used to compare the enriched hallmarks between the two subgroups.

### Statistical analysis

We used GraphPad Prism 8.0 for statistical analysis in this study. Two-sided Student’s *t*-test was used to analyze differences between two groups, while one-way ANOVA was used to compare statistics among three or more groups. We used the *χ*2 test and Fisher’s exact test to analyze the relationship between HIF1A-AS2 expression and clinicopathologic features. Survival analysis was conducted using the Kaplan–Meier method, and we assessed significance using the log-rank test. We considered a *P*-value of < 0.05 to be statistically significant for all tests and the following symbols were used to describe statistical significance: *P < 0.05; **P < 0.01; ***P < 0.001; ns, no significance. The results are presented as mean ± SD of at least three independent experiments.

## Results

### Characterization of HIF1A-AS2 in CRC

**Fig. 1:**
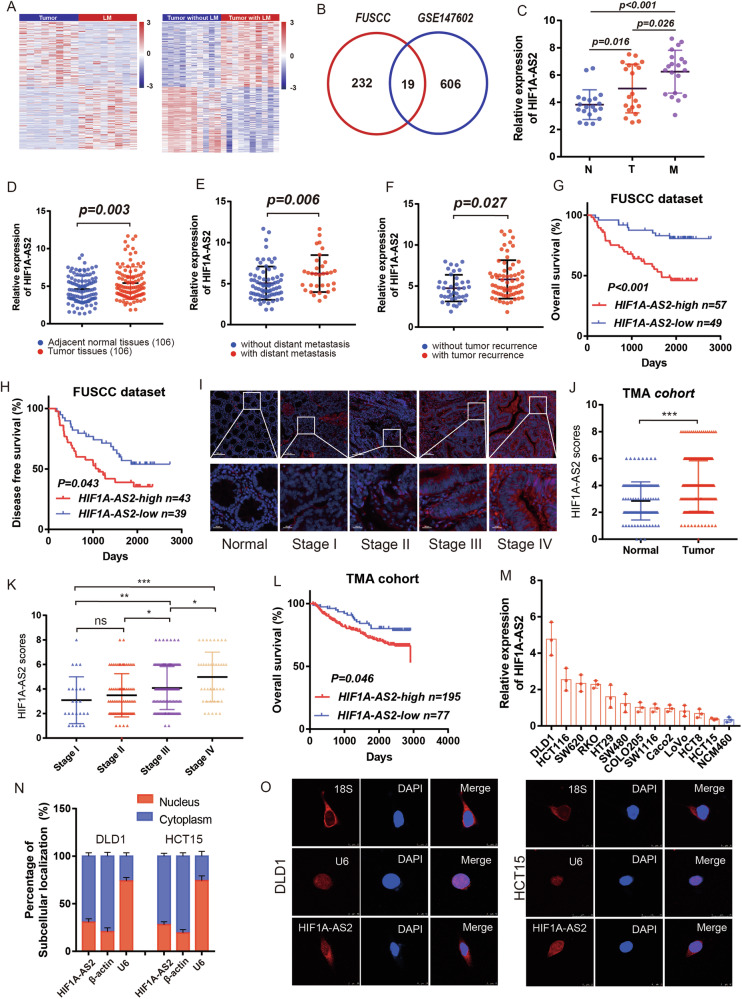
Identification of HIF1A-AS2 as a lncRNA associated with the prognosis and clinicopathological factors of CRC. **A** Heatmaps show some differentially-expressed lncRNAs in the FUSCC CRLM dataset (left) and the GSE147602 dataset (right). **B** Veen plot showing differentially-expressed lncRNAs, which exist in both datasets. **C** Relative HIF1A-AS2 expression in normal tissues, primary CRC samples, and CRLM samples from 20 patients. **D** Relative HIF1A-AS2 expression in CRC tissues and paired normal tissues. **E** Relative HIF1A-AS2 expression in CRC tissues with distant metastasis and those without metastasis. **F** Relative HIF1A-AS2 expression in CRC tissues with recurrence and those without recurrence. **G** Kaplan–Meier curve comparing the OS between the HIF1A-AS2-high group and HIF1A-AS2-low group in the FUSCC cohort. **H** Kaplan–Meier curve comparing the DFS between the HIF1A-AS2-high group and HIF1A-AS2-low group in the FUSCC cohort. **I** Representative FISH images showing HIF1A-AS2 expression in normal and tumor tissues. Scale bar = 100 μm (upper part), 20 μm (lower part). **J** FISH scores of normal and tumor tissues. **K** FISH scores in different TMN stages of CRC. **L** Kaplan–Meier curve comparing the OS between the HIF1A-AS2-high group and HIF1A-AS2-low group in the TMA cohort. **M** Relative HIF1A-AS2 expression in CRC cell lines and NCM460. **N** Relative HIF1A-AS2 expression in subcellular fractions. **O** FISH images showing the subcellular distribution of HIF1A-AS2 in DLD1 (left) and HCT15 cells (right). *P < 0.05; **P < 0.01; ***P < 0.001; ns no significance.

To identify lncRNAs involved in CRC progression and liver metastasis, differentially-expressed lncRNAs (DELs) were analyzed in our own cohort: FUSCC CRLM dataset that contains RNA sequencing data from 8 CRC patients with CRLM. We identified DELs using the following criteria: absolute fold change >1.5 and *P* value < 0.05. We found 251 upregulated lncRNAs in CRLM in the FUSCC CRLM dataset. In order to narrow the screen scope and obtain a more solid result, we introduced another external cohort in our study. GPL21047 is a platform showing lncRNA expression by array and the expression levels of tens of thousands of lncRNAs can be obtained from this platform. GSE147602 dataset, which relies on GPL21047 platform, contains lncRNA expression matrix of 10 primary CRC tissues without liver metastasis and 10 primary CRC tissues with liver metastasis, which was used in this study. By applying the same selection criteria, we found 625 upregulated lncRNAs in primary CRC with LM in GSE147602 dataset. Heatmaps visualized some DELs in the FUSCC and GSE147602 dataset (Fig. 1A). We found 19 lncRNAs were upregulated in both datasets (Fig. 1B), out of which three lncRNAs (HIF1-AS2, LINC00862, and TPRG1-AS1) were previously reported in other studies and selected for further analysis. We experimentally verified the expression of the above three lncRNAs (HIF1-AS2, LINC00862, and TPRG1-AS1) by performing RT-qPCR in 20 pairs of normal-CRC-CRLM samples. HIF1A-AS2 expression increased in adjacent normal tissues, primary CRC tissues, and CRLM tissues, in that order and was therefore identified as a potential oncogenic lncRNA (Figs. 1C and S1A, B). We used both the CPAT dataset and CPC2 to calculate HIF1A-AS2’s coding potential [17, 18]. The results confirmed that HIF1A-AS2 is a non-coding RNA (Fig. S1C, D).

We used a larger cohort containing 106 paired CRC samples with detailed clinicopathologic features (FUSCC cohort) to explore the relationship between HIF1A-AS2 expression and clinicopathologic characters. Our findings revealed that HIF1A-AS2 was upregulated in primacy CRC tissues compared to normal adjacent tissues (Fig. 1D), in CRC tissues with distant metastasis compared to those without distant metastasis (Fig. 1E), and in CRC tissues with tumor recurrence compared to those without tumor recurrence (Fig. 1F). Based on the expression value of HIF1A-AS2, 106 patients were grouped into two groups: HIF1A-AS2-high group (*n* = 57) and HIF1A-AS2-low group (*n* = 49). Our analysis revealed that patients in the HIF1A-AS2-high group had worse overall survival (OS) compared to those in the HIF1A-AS2-low group (Fig. 1G). Additionally, high HIF1A-AS2 level indicated a short disease-free survival (DFS) in 82 patients whose DFS were identified to be greater than 30 days (Fig. 1H). We further adjusted the cutoff value of HIF1-AS2, and our results showed that the HIF1A-AS2-high group had worse OS than the HIF1A-AS2-low group in 31 patients with CRLM (Fig. S1E). Additionally, high HIF1A-AS2 levels in CRLM specimens were associated with shorter OS time in the GSE159216 dataset (Fig. S1F). We next investigated the relationship between HIF1A-AS2 expression and clinicopathological features, and results indicated that HIF1A-AS2 expression was correlated with pathological stage (*P* = 0.026) and metastasis (*P* = 0.009) (Supplementary Table 3). Our univariate and multivariate Cox regression analyses indicated that HIF1A-AS2 expression was significantly correlated with patients’ OS, and high HIF1A-AS2 expression was an independent risk factor (Supplementary Table S7).

We performed a FISH assay to detect HIF1A-AS2 expression in the TMA cohort. A significantly upregulated level of HIF1A-AS2 can be observed in cancerous tissues than in normal samples (Fig. 1I, J). Moreover, high expression of HIF1A-AS2 was correlated with the advanced AJCC stage (Fig. 1K). We categorized patients in the TMA cohort into the HIF1A-AS2-high group (FISH scores range from 3 to 8) and the HIF1A-AS2-low group (FISH scores range from 0 to 2). Similar to the PCR results, high HIF1A-AS2 level also indicated a poor OS in the TMA cohort (Fig. 1L).

To select appropriate CRC cell lines for further investigation, we performed RT-qPCR using 12 CRC cell lines (HCT116, HCT15, HCT8, DLD1, RKO, SW480, SW620, LoVo, HT29, Caco2, COLO205, SW1116), and NCM460. Interestingly, an upregulated expression of HIF1A-AS2 can be observed in all carcinoma cell lines (Fig. 1M). For subsequent experiments, we selected DLD1 and HCT116 cell lines for HIF1-AS2 knockdown and HCT15 and HCT8 cell lines for HIF1A-AS2 overexpression. Subcellular fractionation and FISH assay confirmed that HIF1A-AS2 was predominantly localized in the cytoplasm (Fig. 1N, O).

### HIF1A-AS2 promotes the proliferation, invasion, and metastasis of CRC cells in vitro

**Fig. 2:**
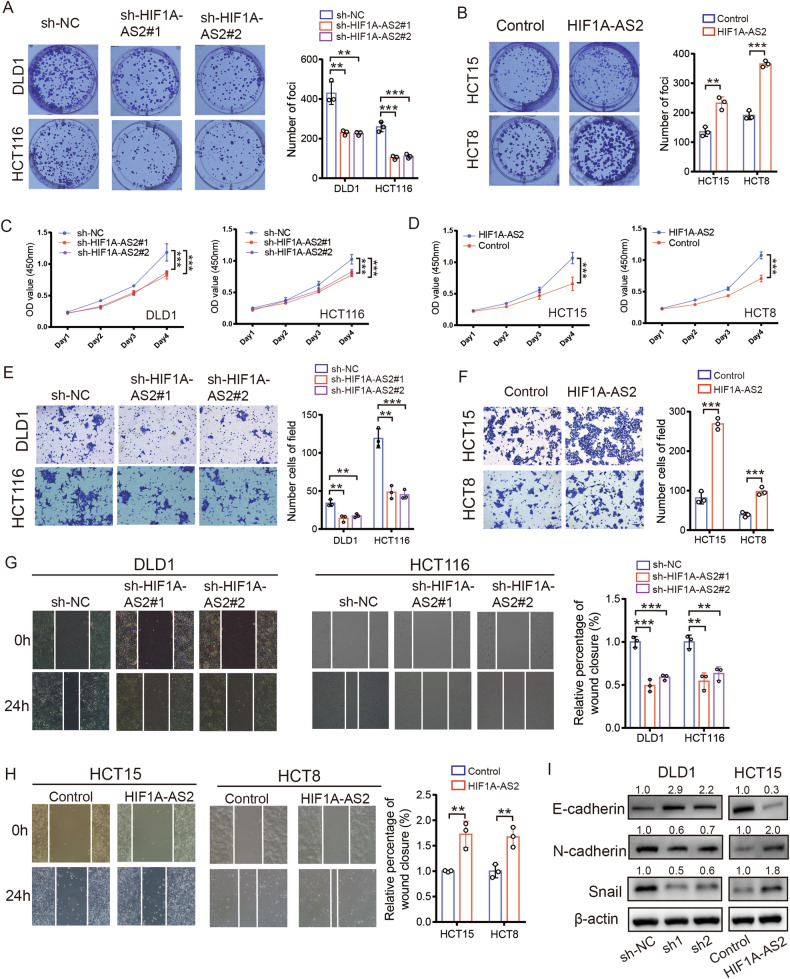
HIF1A-AS2 regulates proliferation, invasion, and metastasis in CRC in vitro. **A**, **B** Colony formation assay of CRC cells after overexpressing or silencing HIF1A-AS2. **C**, **D** The viabilities of CRC cells after overexpressing or silencing HIF1A-AS2. **E**, **F** Transwell invasion assay of CRC cells after overexpressing or silencing HIF1A-AS2. **G**, **H** Wound healing assay showing the migrative ability of CRC cells after overexpressing or silencing HIF1A-AS2. **I** The protein levels of E-cadherin, N-cadherin, and Snail of CRC cells after overexpressing or silencing HIF1A-AS2. *P < 0.05; **P < 0.01; ***P < 0.001; ns no significance.

We generated stable HIF1A-AS2 knockdown and overexpression cell lines, and the transfection efficiency was confirmed (Fig. S2A, B). Results of both colony formation assay and CCK8 assay indicated that HIF1-AS2 inhibition downregulated the proliferative rate of DLD1 and HCT116 cells while HIF1A-AS2 upregulation promoted the proliferative ability of HCT8 and HCT15 cells (Fig. 2A–D). Furthermore, the transwell invasion assay and cell migration assay revealed that knocking down HIF1-AS2 significantly inhibited the metastatic capacity of DLD1 and HCT116 cells, whereas upregulating HIF1A-AS2 enhanced the metastatic ability of HCT8 and HCT15 cells (Fig. 2E–H). Further, HIF1A-AS2 inhibition downregulated the protein levels of mesenchymal markers (Snail, N-cadherin) and promoted the expression of E-cadherin whereas HIF1A-AS2 upregulation did the opposite (Fig. 2I). These experiments showed that HIF1A-AS2 promotes the malignant phenotype of CRC cells in vitro.

### HIF1A-AS2 promotes the proliferation, invasion, and metastasis of CRC cells in vivo

**Fig. 3:**
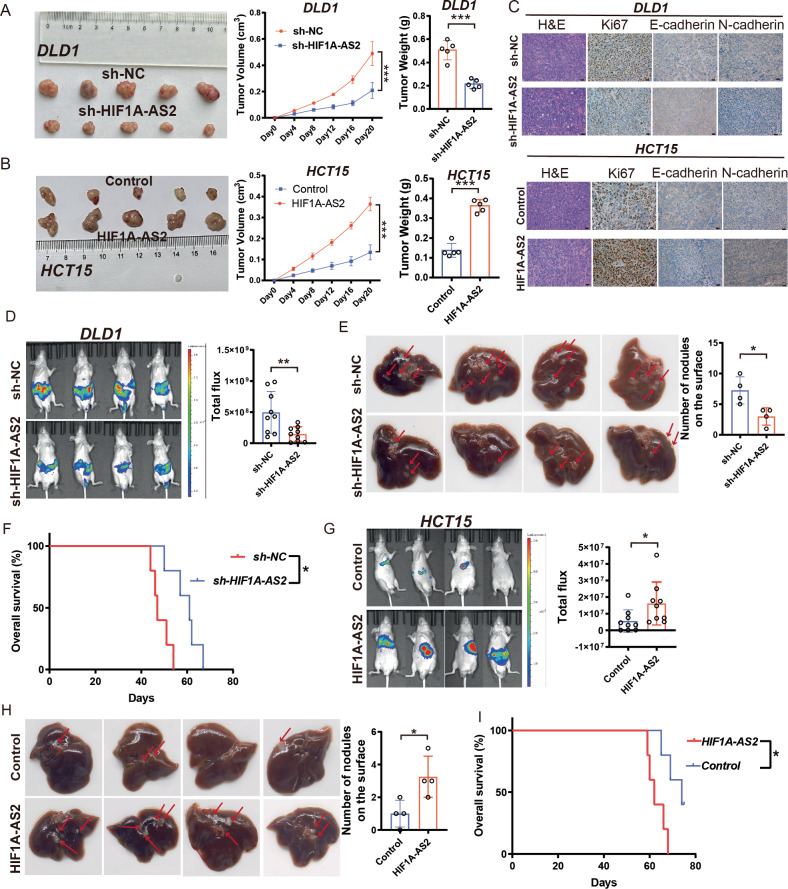
HIF1A-AS2 promotes the proliferation, invasion, and metastasis of CRC cells in vivo. **A**, **B** Images of subcutaneous tumors, Volumes and weights of subcutaneous tumors. **C** Representative IHC images showing the expression of Ki67, E-cadherin, and N-cadherin in subcutaneous tumors in different groups. Scale bar = 20 μm. **D** Representative photographs of bioimaging. **E** Pictures of mouse liver specimen. **F** Kaplan–Meier curve comparing the OS between the sh-NC group and sh-HIF1A-AS2 group. **G** Representative photographs of bioimaging. **H** Pictures of mouse liver specimen. **I** Kaplan–Meier curve comparing the OS between the control group and HIF1A-AS2 group. *P < 0.05; **P < 0.01; ***P < 0.001; ns no significance.

A mouse subcutaneous tumor model was established to evaluate the role of HIF1A-AS2 in tumor growth in vivo. Results demonstrated that HIF1A-AS2 suppression led to a decrease in tumor size and weight, while HIF1A-AS2 overexpression promoted tumor growth (Fig. 3A, B). We collected the xenograft tumor tissues and performed IHC analysis. Notably, the protein levels of Ki67 and N-cadherin were lower, while those of E-cadherin were higher after knocking down HIF1A-AS2. On the contrary, HIF1A-AS2 overexpression promoted the expression of Ki67 and N-cadherin while inhibiting E-cadherin expression (Fig. 3C). Moreover, we established a liver metastasis model using DLD1 cell line (sh-HIF1A-AS2#1, sh-NC) and HCT15 cell line (Control, HIF1A-AS2). The HIF1A-AS2 knockdown significantly reduced the metastatic ability of DLD1 cells, as is revealed by luciferase assay (Fig. 3D). Also, fewer metastatic nodes in the liver were observed in sh-HIF1A-AS2 group compared to the sh-NC group (Fig. 3E). Further, the survival time of mice in sh-NC group was shorter than the survival time of mice in sh-HIF1A-AS2 group (Fig. 3F). On the contrary, overexpressing HIF1A-AS2 in HCT15 cells significantly increased its metastatic ability, as is revealed by luciferase assay result and the number of visible metastatic nodes on the surface of liver (Fig. 3G, H). Additionally, HIF1A-AS2 overexpression significantly shorten the overall survival time of mice in the CRLM model (Fig. 3I). In short, we applied a series of in vivo experiments to demonstrate that HIF1A-AS2 could enhance the proliferative and metastatic ability of CRC cells.

### HIF1A-AS2 promotes aerobic glycolysis in CRC

**Fig. 4:**
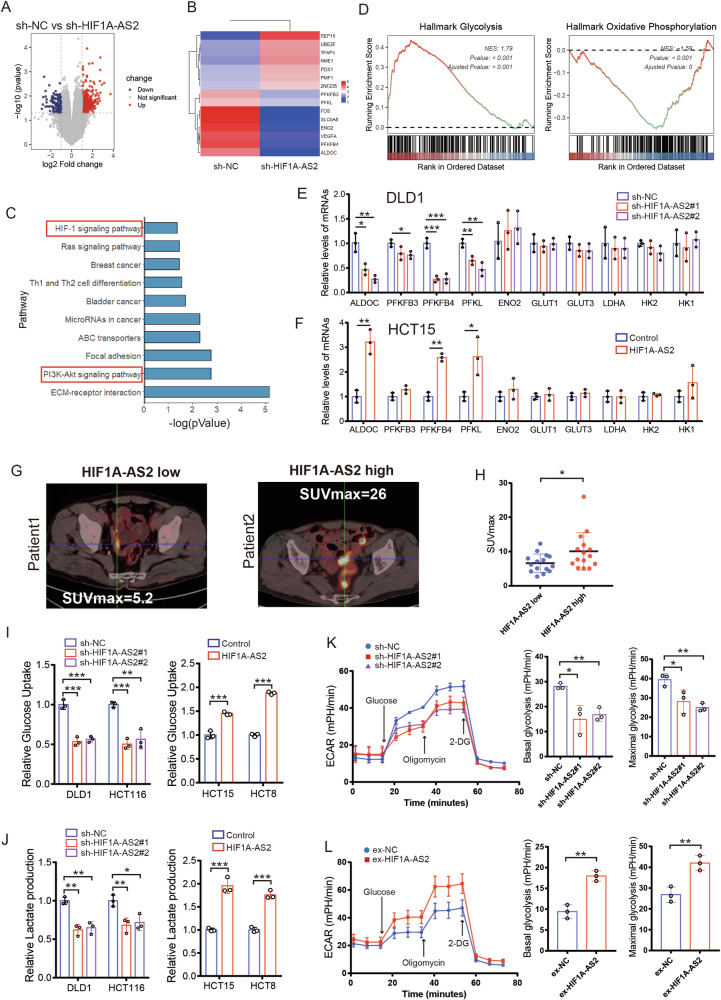
HIF1A-AS2 regulates aerobic glycolysis in CRC. **A** Volcano plot showing the differentially-expressed genes. **B** Heatmap displaying the expression of some differentially-expressed genes. **C** Histogram displaying the significantly enriched pathways of upregulated genes. **D** GSEA results showing the enriched pathways in the sh-NC group. **E** RNA levels of glycolysis-related genes were determined using RT-qPCR in DLD1 cells. **F** RNA levels of glycolysis-related genes were determined using RT-qPCR in HCT15 cells. **G** Representative PET/CT images of the patients in the FUSCC cohort. **H** SUVmax values of the patients undergoing PET/CT examination in the FUSCC cohort. **I** Glucose uptake assay of CRC cells after overexpressing or silencing HIF1A-AS2. **J** Lactate production assay of CRC cells after overexpressing or silencing HIF1A-AS2. **K**, **L** ECAR assay of CRC cells after overexpressing or silencing HIF1A-AS2. *P < 0.05; **P < 0.01; ***P < 0.001; ns no significance.

We performed RNA sequencing using sh-HIF1A-AS2 (*n* = 3) and sh-NC (*n* = 3) DLD1 cell lines to investigate the oncogenic mechanisms of HIF1A-AS2. Differentially-expressed genes were obtained following the criteria of *P* < 0.05 and |Fold change | >1.5, and a volcano plot was created (Fig. 4A). Notably, several glycolysis-related genes, including PFKFB3, PFKFB4, ALDOC, PFKL, and ENO2, were downregulated after HIF1-AS2 knockdown (Fig. 4B). KEGG analysis showed that pathways related to cell adhesion, glycolytic metabolism and cancer are significantly enriched (Fig. 4C). Cell adhesion is related to tumor invasion and metastasis and previous research about HIF1A-AS2 is mainly focused on its role in tumor proliferation and metastasis [19, 20]. However, its role in metabolism remains largely unknown. Hence, we further explored the role of HIF1A-AS2 in the glycolytic metabolism of CRC. Results from GSEA analysis demonstrated that the glycolysis activity of the sh-HIF1A-AS2 group was much lower than that in the sh-NC group, suggesting that HIF1A-AS2 knockdown might inhibited the glycolytic ability of CRC cells (Fig. 4D). Experimentally, we used RT-qPCR to examine the RNA levels of glycolysis-related genes upon HIF1-AS2 knockdown or overexpression. Interestingly, HIF1A-AS2 induced changes in the expression of several metabolic genes, including PFKFB3, PFKFB4, ALDOC, and PFKL (Fig. 4E, F). However, the expression of ENO2, GLUT1, GLUT3, LDHA, HK2, and HK1 remained unchanged. (Fig. 4E, F). Cancer cells often facilitate glucose uptake and glycolysis to support tumor progression [8]. Clinically, the fluoro-2-D-deoxyglucose F18 (18F-FDG) PET/CT has been widely used in malignant tumors not only for initial stage or restage, early treatment response assessment but also for recurrence or metastasis detection and prognosis prediction [21, 22]. The maximum standardized uptake value (SUVmax) of 18F-FDG represents the ability of malignant tumors to uptake glucose, which is an ideal clinical parameter to reflect the glycose uptake ability of tumor. We investigated the relationship between HIF1A-AS2 expression and the SUV max value of CRC tissues. Among the 106 patients in FUSCC dataset, 30 patients performed PET/CT in our center. We next divided patients into high HIF1A-AS2 group (*n* = 15) and low HIF1A-AS2 group (*n* = 15) and compared their SUV max values. Interestingly, higher SUV values can be discovered in the HIF1A-AS2-high group than in its counterpart (Fig. 4G, H). Glucose uptake and lactate production assay demonstrated that HIF1A-AS2 knockdown inhibited glucose intake and lactate formation in DLD1 and HCT116 cells, whereas its overexpression enhanced glucose intake and lactate formation in HCT15 and HCT8 cells (Fig. 4I, J). Additionally, ECAR assay revealed that silencing HIF1A-AS2 suppressed both the basal and maximal glycolytic ability of DLD1 cells while overexpressing HIF1A-AS2 promoted the basal and maximal glycolytic ability of HCT15 cells (Fig. 4K, L).

### HIF1A-AS2 sponges miR-141-3p to downregulate its expression

**Fig. 5:**
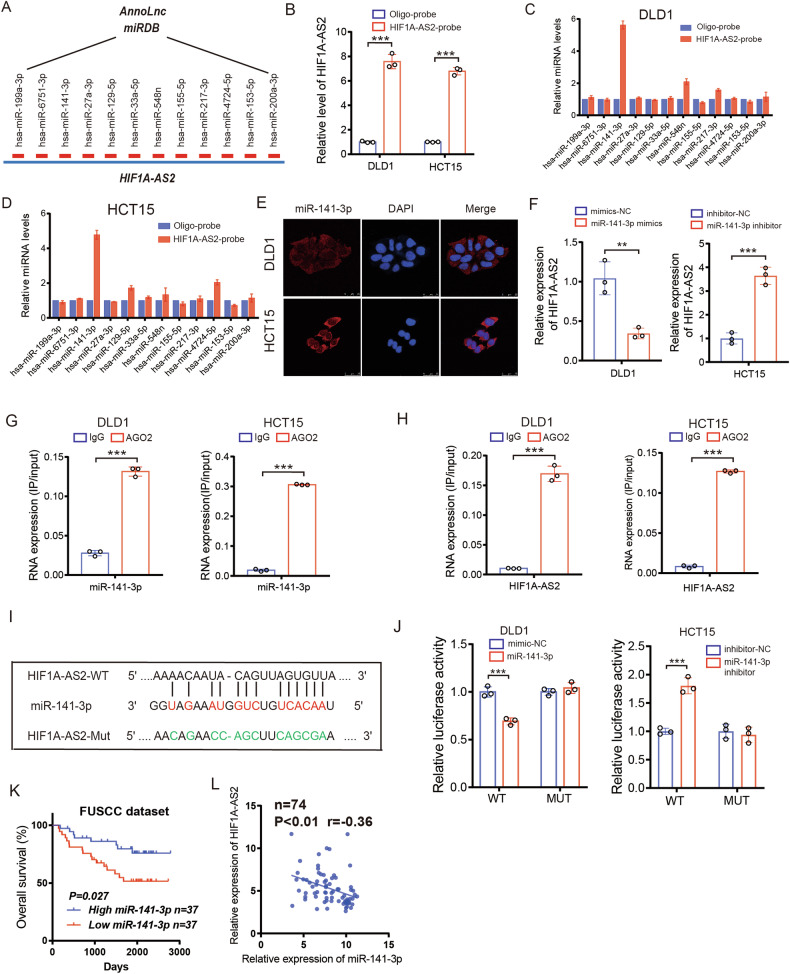
HIF1A-AS2 sponges miR-141-3p to downregulate its expression. **A** Predicted miRNAs that have the potential to bind to HIF1A-AS2. **B** The binding efficiency of HIF1A-AS2 probe and control probe on HIF1A-AS2. **C**, **D** The binding efficiency of HIF1A-AS2 probe and control probe on candidate miRNAs. **E** FISH assay showing the subcellular location of miR-141-3p in CRC cells. Scale bar = 25 μm. **F** HIF1A-AS2 level in CRC cells transfected with miR-141-3p mimics or inhibitor. **G** RIP-qPCR assay was used to determine the binding effect between miR-141-3p and Ago2. **H** RIP-qPCR assay was used to determine the binding effect between HIF1A-AS2 and Ago2. **I** Sequences showing the predicted binding site of HIF1A-AS2 and miR-141-3p and relevant mutant sequences of HIF1A-AS2. **J** Luciferase activity of CRC cells transfected with wild type or mutant HIF1A-AS2 plasmid. **K** Kaplan–Meier curve comparing the OS between miR-141-3p-high group and miR-141-3p -low group in the FUSCC cohort. **L** Correlation between miR-141-3p and HIF1A-AS2 in 74 CRC samples in FUSCC cohort. *P < 0.05; **P < 0.01; ***P < 0.001; ns no significance.

lncRNAs can function as a microRNA sponge to downregulate miRNA expression in the cytoplasm [23]. To investigate whether HIF1A-AS2 could regulate CRC progression by sponging miRNAs, RIP assay was performed, and we found that Ago2 could bind to HIF1A-AS2, suggesting that HIF1A-AS2 can sponge miRNAs via Ago2 complex (Fig. S3A). We utilized the Annolnc and miRDB databases to select 12 potential miRNAs that could bind to HIF1A-AS2 (Fig. 5A). We performed RNA pull-down assays in DLD1 and HCT15 cells using a biotin-labeled probe specifically targeting HIF1A-AS2 and a control probe. RT-qPCR validated the significant binding effect of HIF1A-AS2-specific probe on HIF1A-AS2 (Fig. 5B). Further, only miR-141-3p was markedly pulled down by the HIF1A-AS2-specific probe among the 12 candidate miRNAs in both cell lines, as revealed by RT-qPCR, indicating a specific interaction between miR-141-3p and HIF1A-AS2 (Fig. 5C, D). FISH assays revealed the subcellular location of miR-141-3p, suggesting that HIF1A-AS2 interacts with miR-141-3p in the cytoplasm (Fig. 5E). A lower miR-141-3p level can be found in CRC tissues compared to normal tissues, as revealed by GSE41655 and GSE48267 datasets (Fig. S3B, C). Additionally, miR-141-3p mimics could downregulate HIF1A-AS2 expression, whereas miR-141-3p inhibitor could promote its expression (Fig. 5F). RIP assay confirmed that both miR-141-3p and HIF1A-AS2 were enriched in Ago2-mediated immunoprecipitation, suggesting that Ago2 complex involves in the binding of miR-141-3p and HIF1A-AS2 (Fig. 5G, H). We next inserted the wild-type HIF1A-AS2 sequence (HIF1A-AS2-WT) or HIF1A-AS2 sequence with a mutated miR-141-3p binding site (HIF1A-AS2-Mut) into pmirGLO plasmids, which were then co-transfected with miR-141-3p mimics or inhibitors (Fig. 5I). Interestingly, miR-141-3p mimics markedly downregulated the luciferase activity of HIF1A-AS2-WT plasmid while miR-141-3p inhibitors significantly increased the luciferase activity of HIF1A-AS2-WT plasmid (Fig. 5J). However, they showed no inhibitory effect on the luciferase activity of HIF1A-AS2-Mut plasmid in both DLD1 and HCT15 cells (Fig. 5J). We also divided 74 patients into the miR-141-3p-high group (*n* = 37), and miR-141-3p-low group (*n* = 37), and survival analysis demonstrated that the miR-141-3p-low group had shorter OS than the miR-141-3p-high group (Fig. 5K). Moreover, we quantified the miR-141-3p level in 74 CRC patients and discovered a negative correlation between HIF1A-AS2 and miR-141-3p (Fig. 5L). In summary, these findings suggest that HIF1A-AS2 can sponge miR-141-3p to downregulate its expression in CRC.

### miR-141-3p binds to the 3’UTR of FOXC1 to downregulate its expression

**Fig. 6:**
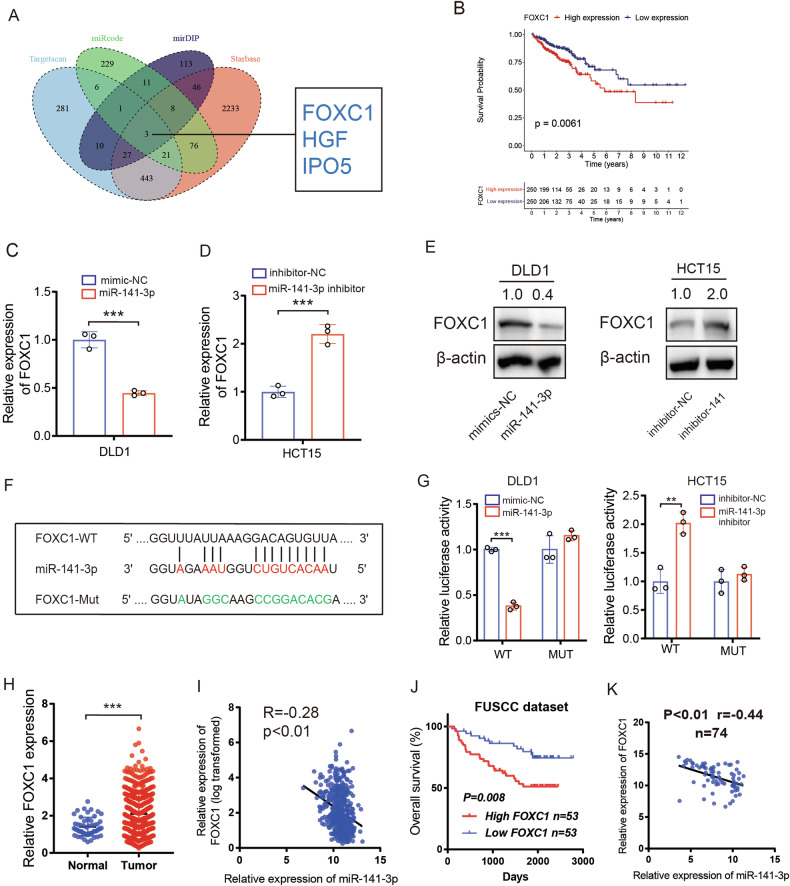
miR-141-3p binds to the 3’UTR of FOXC1 to downregulate its expression. **A** Veen plot displaying the predicted mRNAs by four datasets. **B** Kaplan–Meier curve comparing the OS between FOXC1-high group and FOXC1-low group in TCGA cohort. **C** RT-qPCR showing the expression of FOXC1 after transfecting CRC cells with miR-141-3p mimics. **D** RT-qPCR showing the expression of FOXC1 after transfecting CRC cells with miR-141-3p inhibitors. **E** Western blot shows the expression of FOXC1 after transfecting CRC cells with miR-141-3p mimics or inhibitor. **F** Sequences showing the predicted binding site between the 3’UTR of FOXC1 and miR-141-3p and relevant mutant sequences of the 3’UTR of FOXC1. **G** Luciferase activity of CRC cells transfected with wild type or mutant FOXC1 3’UTR plasmid. **H** Relative FOXC1 mRNA levels of normal and tumor samples in TCGA cohort. **I** Correlation between miR-141-3p and FOXC1 mRNA level in TCGA cohort. **J** Kaplan–Meier curve comparing the OS between the FOXC1-high group and FOXC1-low group in the FUSCC cohort. **K** Correlation between miR-141-3p and FOXC1 in 74 CRC samples in the FUSCC cohort. *P < 0.05; **P < 0.01; ***P < 0.001; ns no significance.

We used Starbase, miRcode, mirDIP, and Targetscan database to investigate the downstream targets regulated by miR-141-3p and obtained FOXC1, HGF, and IPO5 as potential candidates (Fig. 6A). Survival analysis revealed that high levels of FOXC1 indicated a worse prognosis in CRC patients, while high levels of HGF and IPO5 did not suggest a poor prognosis according to the TCGA cohort (Figs. 6B and S4A, B). We next transfected DLD1 cells with miR-141-3p mimics and used RT-qPCR to detect the mRNA level of FOXC1, HGF, and IPO5. Interestingly, only the mRNA level of FOXC1 was regulated by miR-141-3p mimics (Figs. 6C and S4C, D). We also found an upregulated level of FOXC1 in HCT15 cells that were transfected with miR-141-3p inhibitor (Fig. 6D). Thus, FOXC1 was selected as the downstream target of miR-141-3p. We further verified the regulatory role of miR-141-3p on FOXC1 using western blot analysis (Fig. 6E). To find out whether miR-141-3p could bind to the 3’UTR of FOXC1, we mutated the predicted binding sequence between miR-141-3p and 3’UTR of FOXC1 and inserted the mutant or wild-type sequence into the pmirGLO plasmid (FOXC1-WT, FOXC1-Mut) (Fig. 6F). Interestingly, the luciferase activity of FOXC1-WT can be markedly downregulated by miR-141-3p mimics in DLD1 cells and upregulated by miR-141-3p inhibitors in HCT15 cells, while the luciferase activity of FOXC1-Mut was not influenced by miR-141-3p mimics or inhibitors (Fig. 6G). In the TCGA CRC cohort, FOXC1 expression was much higher in cancerous tissues than in normal tissues, and it had a significantly negative correlation with miR-141-3p expression (Fig. 6H, I). The FUSCC cohort verified that patients in the FOXC1-high group (*n* = 53) had a worse OS compared to patients in the FOXC1-low group (*n* = 53) (Fig. 6J). Furthermore, a negative correlation between miR-141-3p and FOXC1 was also discovered in our cohort (Fig. 6K). Collectively, we discovered that miR-141-3p can bind to the 3’UTR of FOXC1 to downregulate its expression.

### HIF1A-AS2/miR-141-3p/FOXC1 axis regulates the progression of CRC

**Fig. 7:**
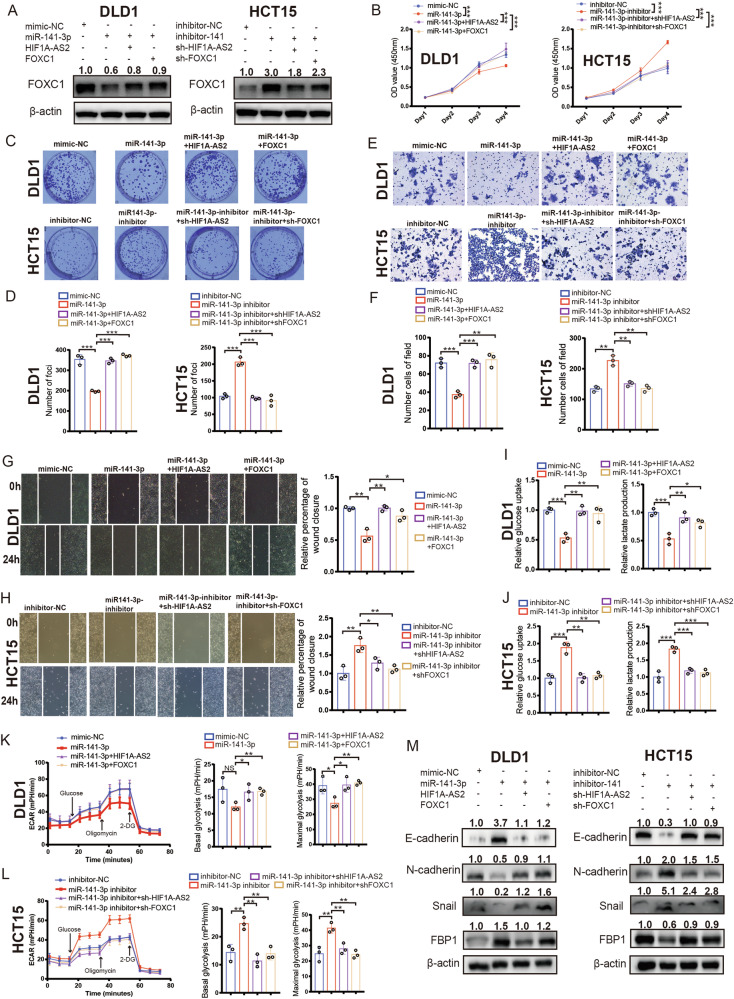
HIF1A-AS2/miR-141-3p/FOXC1 axis regulates CRC progression. **A** Western blot showing the protein level of FOXC1 in CRC cells with different treatments. **B** The viabilities of CRC cells with indicated treatments. **C**, **D** Colony formation assay of CRC cells with indicated treatments. **E**, **F** The invasive ability of CRC cells with indicated treatments were measured using transwell invasion assay. **G**, **H** The migratory ability of CRC cells with indicated treatments were measured using cell migration assay. **I**, **L** Glucose uptake assay, lactate production assay, and ECAR assay of CRC cells with relevant treatments. **M** Protein levels of E-cadherin, N-cadherin, Snail, and FBP1 of CRC cells with indicated treatments. *P < 0.05; **P < 0.01; ***P < 0.001; ns no significance.

FOXC1 regulates proliferation, metastasis, and glycolysis in multiple cancers [24, 25]. We treated DLD1 and HCT15 using different methods to investigate whether HIF1A-AS2 could regulate CRC progression via miR-141-3p/FOXC1 axis. The inhibitory role of miR-141-3p-mimic on FOXC1 expression was diminished when HIF1A-AS2 or FOXC1 were overexpressed, and the stimulatory effect of the miR-141-3p-inhibitor on FOXC1 expression could be reversed by knocking down HIF1A-AS2 or FOXC1 (Fig. 7A). These findings suggest that FOXC1 expression can be regulated via the HIF1A-AS2/miR-141-3p axis (Fig. 7A). Functional experiments demonstrated that miR-141-3p could suppress cell proliferation and metastasis, which could be restored by overexpressing HIF1A-AS2 or FOXC1 (Fig. 7B–H). In contrast, inhibition of miR-141-3p promoted cell proliferation and metastasis, which could be abolished by silencing HIF1A-AS2 or FOXC1 (Fig. 7B–H). Western blot assay also suggested that HIF1A-AS2/miR-141-3p/FOXC1 axis could regulate the expression of EMT markers (Fig. 7M). Our previous research demonstrated that FOXC1 inhibits FBP1 expression to promote glycolysis in CRC [24]. We performed a glucose uptake assay, lactate production assay, and ECAR assay to see whether HIF1A-AS2/miR-141-3p/FOXC1 axis could regulate glycolysis. Interestingly, miR-141-3p suppressed the glycolytic ability of DLD1 cells, which could be abolished by overexpressing FOXC1 or HIF1A-AS2 (Fig. 7I–L). In contrast, miR-141-3p inhibitor could enhance the glycolytic level of HCT15 cells, and this could be reversed by FOXC1 or HIF1A-AS2 knockdown (Fig. 7I–L). Moreover, we also found that HIF1A-AS2 could negatively regulate FBP1 expression via miR-141-3p/FOXC1 axis (Fig. 7M). In addition, a significant positive correlation can be observed between HIF1A-AS2 and FOXC1 expression (Fig. S5). Collectively, these findings demonstrate that HIF1A-AS2 exerts its oncogenic functions via the miR-141-3p/FOXC1 axis in CRC.

### HIF1A-AS2 is transcriptionally activated by SP1 in CRC cells

**Fig. 8:**
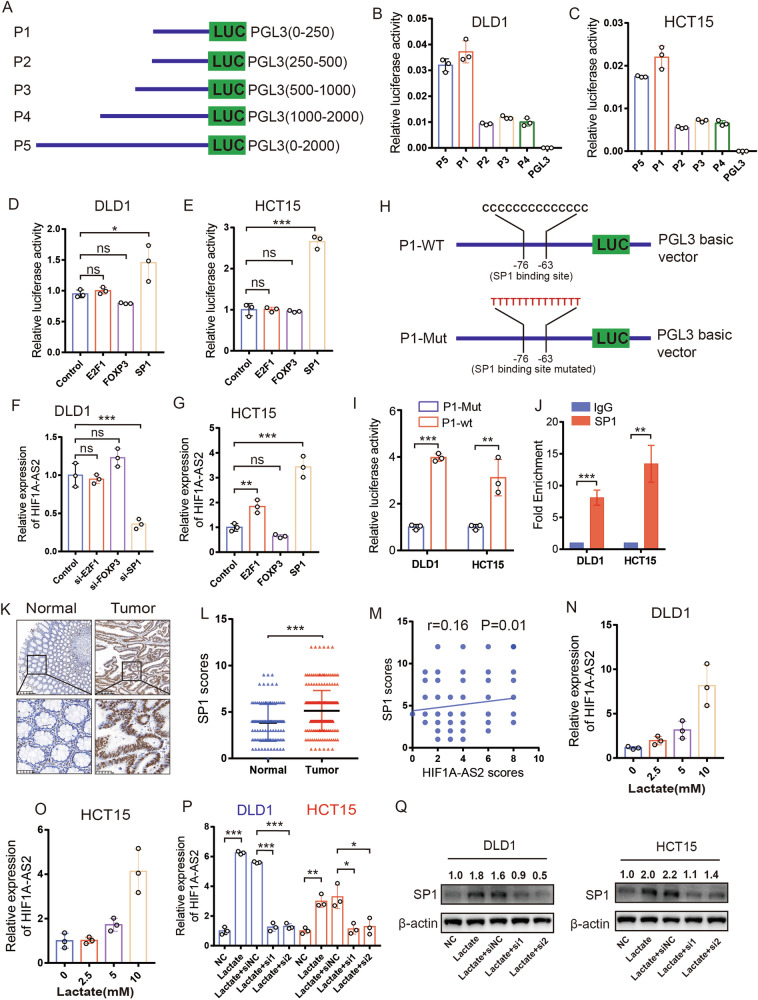
SP1 transcriptionally activates HIF1A-AS2 in CRC. **A** The fragments of HIF1A-AS2 promoter were cloned into the PGL3 plasmid. **B**, **C** Relative luciferase activity of CRC cells transfected with the relevant fragment of HIF1A-AS2 promoter. **D**, **E** Luciferase activity assay showing the transcriptional activity of HIF1A-AS2 promoter in CRC cells with indicated treatments. **F** Relative HIF1A-AS2 level of CRC cells with different treatments in DLD1 cells. **G** Relative HIF1A-AS2 level of CRC cells with different treatments in HCT15 cells. **H** Predicted binding site of SP1 on HIF1A-AS2 and relevant mutant sequence. **I** Relative luciferase activity of CRC cells transfected with wild-type or mutant HIF1A-AS2 promoter. **J** CHIP-qPCR displaying the interaction of SP1 and HIF1A-AS2 promoter. **K** Representative IHC images showing SP1 expression in normal and tumor tissues. Scale bar = 200 μm (upper part), 50 μm (lower part). **L** SP1 scores of normal and tumor tissues in TMA cohort. **M** Correlation between SP1 score and HIF1A-AS2 score in TMA cohort. **N**, **O** Relative HIF1A-AS2 expression in CRC cells treated with different concentrations of lactate. **P** Relative HIF1A-AS2 expression in CRC cells with relevant treatments. **Q** Western blot showing SP1 expression in CRC cells with relevant treatments. *P < 0.05; **P < 0.01; ***P < 0.001; ns no significance.

The promoter sequence (2000bp) of HIF1A-AS2 was obtained by searching the Ensembl Genome Browser online database (http://asia.ensembl.org/index.html). To find out the promoter region having the highest transcriptional activity, the 2000bp promoter sequence was divided into four fragments. These fragments as well as the full-length promoter (2000bp) were inserted into pGL3-basic vector (named PGL3(0–250)/P1, PGL3(250–500)/P2, PGL3(500–1000)/P3, PGL3(1000–2000)/P4 and PGL3(0–2000)/P5, respectively) (Fig. 8A). P1/PGL3(0–250) exhibited the highest transcriptional activity among the four fragments, and its transcriptional activity was similar to that of P5/PGL3(0–2000) in both DLD1 and HCT15 cell lines, suggesting that the upstream 250 bp region was essential for HIF1A-AS2 transcription (Fig. 8B, C). JASPAR (http://jaspar.genereg.net/) and PROMO (http://alggen.lsi.upc.es/cgi-bin/promo_v3/promo/promoinit.cgi?dirDB=TF_6.4) datasets were used to discover TFs binding to the upstream 250 bp promoter sequence. Three TFs (FOXP3, E2F1, and SP1) were selected for experimental verification. Luciferase assay showed that only SP1 overexpression could enhance the transcriptional activity of P1 (Fig. 8D, E). Meanwhile, RT-qPCR results indicated that SP1 knockdown in DLD1 cells could downregulate HIF1A-AS2 expression, while SP1 overexpression had the opposite effect (Fig. 8F, G). This phenomenon was also observed in HCT116 and HCT8 cells (Fig. S6A, B). We mutated the predicted SP1 binding site of the P1 promoter and then overexpressed SP1 in CRC cells to evaluate the transcriptional activity of the P1-Mut promoter and P1-WT promoter (Fig. 8H). Interestingly, the transcriptional activity of the P1-Mut promoter was significantly lower than that of the P1-WT promoter (Fig. 8I). ChIP-qPCR analysis confirmed the interaction between SP1 and HIF1A-AS2 promoter (Fig. 8J). Results from the TMA cohort also suggested the protein levels of SP1 were upregulated in tumor samples (Fig. 8K, L). Furthermore, the HIF1A-AS2 scores from FISH analysis showed a positive correlation with SP1 scores in the TMA cohort (Fig. 8M). Taken together, we found that SP1 promotes the transcription of HIF1A-AS2.

The extracellular microenvironment has been reported to alter the lncRNA expression [26]. We cultured cells for 0 h, 12 h, 24 h, or 48 h without changing the culture medium. Interestingly, HIF1A-AS2 expression increased in a time-dependent manner (Fig. S6C). To test the hypothesis that lactate might regulate HIF1A-AS2 expression, we cultured CRC cells in culture media with different concentrations of lactate for 12 h. We found lactate could upregulate HIF1A-AS2 expression in a concentration-dependent manner (Fig. 8N, O). Furthermore, lactate could increase the protein level of SP1, and lactate-induced HIF1A-AS2 upregulation was significantly inhibited after knocking down SP1 (Fig. 8P, Q). Hence, lactate could stimulate SP1 expression to promote HIF1A-AS2 transcription.

### Exosomal HIF1A-AS2 enhances proliferation, metastasis, and glycolysis in CRC cells

**Fig. 9:**
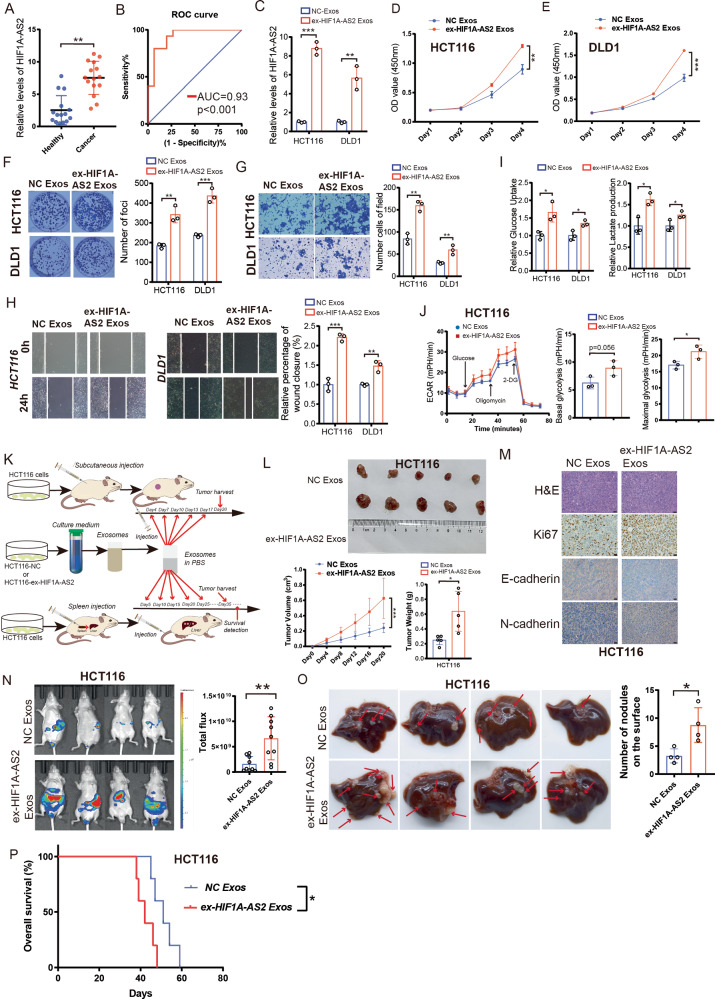
Exosomal HIF1A-AS2 can promote CRC progression. **A** Relative exosomal HIF1A-AS2 expression in the plasma of 15 healthy people and 15 CRC patients. **B** ROC curve showing the diagnostic potential of exosomal HIF1A-AS2. **C** Relative exosomal HIF1A-AS2 level after overexpressing HIF1A-AS2 in CRC cells. **D**, **E** The viabilities of CRC cells with relative treatments. **F** Colony formation assay of CRC cells with relative treatments. **G**, **H** Transwell invasion assay and cell migration assay showing the metastatic ability of CRC cells with relative treatments. **I**, **J** Glucose uptake assay, lactate production assay, and ECAR assay of CRC cells with relevant treatments. **K** Cartoon displaying the process about how the exosome was extracted from the supernatant and injected into mice. **L** Images of subcutaneous tumors, volumes of subcutaneous tumors and weights of subcutaneous tumors. **M** Representative IHC images showing the expression of Ki67, E-cadherin, and N-cadherin in subcutaneous tumors in different groups. Scale bar = 20 μm. **N** Representative photographs of bioimaging. **O** Pictures of mouse liver specimen. **P** Kaplan–Meier curve comparing the OS between the sh-NC group and sh-HIF1A-AS2 group.

Exosomal lncRNAs widely take part in the progression of malignant tumors and are highly enriched in biological fluids, which can be used for liquid biopsies [27]. We extracted exosomes from the serum of 15 healthy subjects and 15 CRC patients and performed RT-qPCR to see whether exosomal HIF1A-AS2 was abnormally overexpressed in the plasma of CRC patients. Interestingly, the exosomal HIF1A-AS2 level was significantly higher in the serum of CRC patients than in normal people, with an area under the receiver operating characteristic (ROC) curve of 0.93, suggesting that exosomal HIF1A-AS2 might take part in the progression of CRC and can function as a diagnostic marker (Fig. 9A, B).

We overexpressed HIF1A-AS2 in DLD1 and HCT116 cells and isolated exosomes from the supernatant of DLD1 and HCT116 cells using ultracentrifuge method. Exosomes derived from ex-HIF1A-AS2 CRC cells (ex-HIF1A-AS2 Exos) exhibited higher expression of HIF1A-AS2 compared to the control group (NC Exos) (Fig. 9C). We validated the morphologies and sizes of the exosomes from four groups (HCT116-NC, HCT116-OE, DLD1-NC, and DLD1-OE) using a transmission electron microscope (Fig. S7A). The size and number of tumor-derived exosomes were determined using nanoparticle tracking analysis (NTA) (Fig. S7B, C). Moreover, exosomal markers, including TSG101, HSP70, and Annexin A1, were validated by western blot (Fig. S7D). We performed fluorescence dye-staining of exosomes and found that exosomes were endocytosed by CRC cells (Fig. S7E). The exosomal HIF1A-AS2 level remained stable for 12 h in both HCT116 and DLD1 cells (Fig. S7F).

Previous research has reported that HIF1A-AS2 was able to regulate Glioblastoma multiforme (GBM) progression and radiotherapy via exosomes [28]. We next explored whether exosomal HIF1A-AS2 is involved in CRC progression. We co-cultured tumor-derived exosomes with HCT116 and DLD1 cells. Colony formation assay and CCK8 assay confirmed that CRC cells treated with ex-HIF1A-AS2 exosomes had higher proliferation than that in cells treated with NC exosomes (Fig. 9D–F). Transwell invasion assay and cell migration assay confirmed that exosomal HIF1A-AS2 could promote the metastatic ability of CRC cells (Fig. 9G, H). Furthermore, our experiments revealed that ex-HIF1A-AS2 exosomes also promoted the lactate production and glucose uptake of CRC cells (Fig. 9I). The results from the ECAR assay further corroborated the stimulatory effect of exosomal HIF1A-AS2 on aerobic glycolysis in CRC (Fig. 9J).

To find out whether exosomal HIF1A-AS2 promotes tumor growth and metastasis in vivo, HCT116-derived exosomes were injected into the tail vein of the subcutaneous tumor model or CRLM model mice (Fig. 9K). The subcutaneous tumors in the ex-HIF1A-AS2 Exos group had higher growth rates and weights (Fig. 9L). IHC staining confirmed that the tumors in the ex-HIF1A-AS2 Exos group had higher Ki67 and N-cadherin expression and lower E-cadherin expression compared to those in the ex-NC Exos group (Fig. 9M). Furthermore, mice treated with ex-HIF1A-AS2 Exos exhibited a significantly higher fluorescence intensity and higher number of metastatic nodes in the liver than those in mice treated with ex-NC Exos, with shorter survival time comparing to control group (Fig. 9N–P).

Taken together, these data suggested that tumor-derived exosomal HIF1A-AS2 could promote the progression of CRC.

## Discussion

**Fig. 10:**
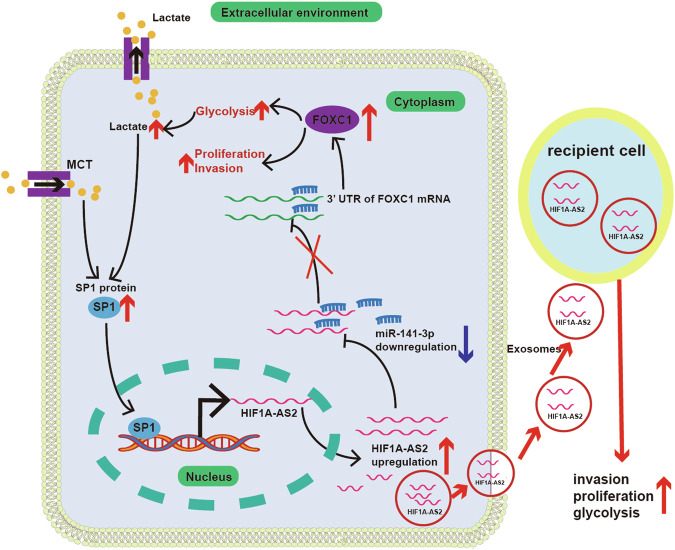
Flow chart of this research. SP1 can transcriptionally activate HIF1A-AS2. HIF1A-AS2 can promote FOXC1 expression by sponging miR-141-3p. Further, HIF1A-AS2 can be packaged into exosomes and promote the malignant phenotype of recipient tumor cells.

Although recent advances in cancer therapy have improved patients’ outcomes, it is predicted that over 50,000 people will die from CRC in the USA in 2023 [29]. The development of liver metastasis contributes to the majority of CRC-related death [30]. Therefore, finding effective methods to prevent or treat CRLM is imperative. Cancer metabolism has distinct features that differ from those of normal cells, making them promising therapeutic targets in CRC [3]. Aerobic glycolysis, which is not a metabolic feature of normal colon epithelial cells, is universally present in CRC cells [8]. Emerging research has shown the evidence that lncRNAs could impact the glycolytic metabolism of CRC [31, 32]. For example, lncRNA GLCC1 stabilizes c-Myc, whose upregulation promotes the transcription of glycolytic genes and glycolytic metabolism activation in CRC [31]. Through bioinformatic analysis and experiments, we discovered that SP1-activated lncRNA HIF1A-AS2 can accelerate CRC progression by promoting the mRNA level of FOXC1 (Fig. 10).

RNA sequencing data from 8 primary CRC tissues and paired CRLM tissues was analyzed, and an abnormal upregulation of HIF1A-AS2 was discovered in CRLM samples. Additionally, we experimentally validated that HIF1A-AS2 is upregulated in adjacent normal tissues, primary CRC tissues, and CRLM tissues in order, suggesting its potential involvement in CRC progression. We evaluated the association of HIF1A-AS2 with clinicopathological factors in two independent cohorts: the FUSCC cohort (cDNA from 106 CRC samples and paired normal samples) and; the TMA cohort (paraffin sections of 272 CRC samples). Results from both cohorts suggested that HIF1A-AS2 is overexpressed in CRC tissues, and high HIF1A-AS2 level correlates to poor prognosis and advanced stage in CRC patients. Although functional experiments confirmed that HIF1A-AS2 could promote CRC progression, the regulatory mechanisms are largely unknown. Using RNA sequencing, we analyzed the pathways affected by HIF1A-AS2 knockdown and found that HIF1A-AS2 might participate in regulating the glycolytic activity of CRC cells. Glucose uptake assay, lactate production assay, and ECAR assay further validated that HIF1A-AS2 can regulate glycolytic metabolism in CRC.

By sponging miRNAs, binding to proteins or mRNAs to regulate their expression, encoding small peptides, and so on, lncRNAs widely involve in gene regulation [9]. The most well-studied function of lncRNA is the sponging of miRNA to indirectly regulate miRNA-targeted genes [23, 33]. By searching online databases and performing relevant experiments, we found that HIF1A-AS2 can sponge miR-141-3p to indirectly promote FOXC1 expression. miR-141-3p is found to suppress the progression of multiple cancers [34–36]. Similar to previous research, our functional analysis revealed that miR-141-3p inhibits the proliferation, metastasis, and aerobic glycolysis of CRC cells. Additionally, our results indicated that patients with high miR-141-3p expression have better OS than those with low miR-141-3p expression. FOXC1 is a well-known oncogene that drives tumorigenesis in multiple cancers [25]. Our previous research suggested that FOXC1 can promote the proliferation and aerobic glycolysis of CRC cells by inhibiting FBP1 transcription [24]. In this study, we discovered that FOXC1 levels are negatively correlated with patients’ survival and HIF1A-AS2 expression. Further, FOXC1 can restore the proliferative, invasive, migrative, and glycolytic ability of CRC cells transfected with miR-141-3p mimics. Conversely, the malignant phenotype of CRC cells transfected with miR-141-3p inhibitor can be reversed after knocking down FOXC1.

The mechanism through which HIF1A-AS2 expression can be upregulated is still largely unknown. Multiple studies have shown that some transcriptional factors can promote or inhibit the transcription of lncRNAs [37, 38]. In this study, we first showed that the upstream 250 bp promoter sequence of HIF1A-AS2 has the highest transcriptional activity. Then, we found that SP1 binds to the upstream 250 bp promoter sequence of HIF1A-AS2 and promotes its transcription. SP1 is found to involve in promoting the transcription of several lncRNAs [39, 40]. Sp1 can bind to putative CG-rich Sp-binding sites in the promoter region of genes to promote their transcription [41]. JASPAR database predicted the binding site between SP1 and the P1 fragment. Mutation of the binding site in the P1 fragment significantly reduced its luciferase activity. SP1 expression is higher in tumor tissues than in normal tissues, and the protein levels of SP1 have a positive correlation with HIF1A-AS2 levels, demonstrating that SP1 transcriptionally activates HIF1A-AS2. Interestingly, HIF1A-AS2-induced lactate could, in turn, promotes SP1 expression to ulteriorly promote HIF1A-AS2 transcription, forming a positive feedback loop that is essential to CRC progression.

Exosomes function as carriers of DNA, RNA, and proteins and are important mediators of intercellular communication in CRC [42, 43]. Some exosomal lncRNAs can contribute to tumor metastasis, drug resistance, and an immune-suppressive microenvironment [27, 44, 45]. HIF1A-AS2 was previously reported to be packaged into exosomes, and exosomal HIF1A-AS2 could promote the viability, invasion, and radio-resistance of recipient glioblastoma multiforme (GBM) cells [28]. Therefore, we hypothesized that HIF1A-AS2 is packaged into exosomes to promote the malignant phenotype of recipient CRC cells. We first detected exosomal HIF1A-AS2 expression using the plasma of 15 CRC patients and 15 healthy controls. Our experiments suggested that exosomal HIF1A-AS2 expression is significantly higher in the plasma of CRC patients than that in healthy controls, with an AUC value of 0.93, suggesting that serum exosomal HIF1A-AS2 can be a promising diagnostic marker for CRC. Experimentally, exosomal HIF1A-AS2 can promote the proliferation, metastasis, and glycolysis of recipient CRC cells. Hence, we concluded that exosomal HIF1A-AS2 can be utilized as a diagnostic marker and regarded as a potential target in CRC.

## Conclusion

HIF1A-AS2 is abnormally upregulated and related to clinicopathological factors in CRC. HIF1A-AS2 could promote the proliferation, metastasis, and aerobic glycolysis of CRC cells by sponging miR-141-3p to enhance FOXC1 expression. The increased extracellular lactate concentration could, in turn, enhance SP1 expression, forming a positive feedback loop. Further, our analyses reveal that HIF1A-AS2 is packaged into exosomes to promote the malignant phenotype of recipient cells. Extracellular lactate could upregulate the expression of SP1, which could further activate the transcription of HIF1A-AS2. Our study highlights the potential of HIF1A-AS2 as a diagnostic biomarker and therapeutic target in CRC.

## Supplementary information

Supplementary material

original data

## Data Availability

In this study, the datasets supporting the conclusions of this article are included within the article and its additional files.
