# Supplementary material for: HIF1A-AS2 promotes the metabolic reprogramming and progression of colorectal cancer via miR-141-3p/FOXC1 axis
Source: Cell Death Dis. 2024 Sep 3;15(9):645. doi: 10.1038/s41419-024-06958-2 (PMC11372083; doi:10.1038/s41419-024-06958-2)
Supplement: Supplementary file 1 — Supplementary material [file 41419_2024_6958_MOESM1_ESM.pdf]

Supplementary information

Table S1:The sequences of the siRNAs and shRNAs	Sequence(5’-3’)
sh-HIF1A-AS2#1	AAGCTGATCAAAGGGGCCTGGTC
sh-HIF1A-AS2#2	GAGTTGGAGGTGTTGAAGCAAAT
sh-FOXC1	GTCACAGAGGATCGGCTTGAA
sh-NC	GATCCGCTCTGGCACTCAGAATAA
si-SP1#1	CACUUAUUCCCUCAUCCUUTT
si-SP1#2	GCAAGUUCUGACAGGACUACCTT
si-NC	UUCUCCGAACGUGUCACGUTT

Table S2:The sequences of the miRNA mimics and inhibitors	Sequence(5’-3’)
miR-141-3p sense	UAACACUGUCUGGUAAAGAUGG
miR-141-3p anti-sense	CCAUCUUUACCAGACAGUGUUA
miR-NC sense	UUUGUACUACACAAAAGUACUG
miR-NC anti-sense	AAACAUGAUGUGUUUUCAUGAC
miR-141-3p inhibitor	CCAUCUUUACCAGACAGUGUUA
inhibitor NC	CAGUACUUUUGUGUAGUACAAA

Table S3:The clinicopathological features of patients in FUSCC cohort

Clinicopathologic parameters	Case	HIF1A-AS2 expression		*P value*
	(n = 106)	Low	High	
Total	106	49	57	
Gender				0.094
Male	69	36	33	
Female	37	13	24	
Age				0.596
≥ 60	62	30	32	
< 60	44	19	25	
Pathological stage				**0.026***
I	9	7	2	
II	21	10	11	
III	43	23	20	
IV	33	9	24	
T stage				0.169
T1-2	11	8	3	
T3	48	20	28	

T4	47	21	26	
Lymph node metastasis				0.247
N0	35	18	17	
N1	41	21	20	
N2	30	10	20	
Distant metastasis				**0.009****
M0	73	40	33	
M1	33	9	24	

Table S4: TMN stage of the patients in TMA cohort			
Factors		Case	Proportion
T stage			
	T1	3	1.10%
	T2	42	15.40%
	T3	49	18.00%
	T4	178	65.40%
N stage			
	N0	113	41.50%
	N1	89	32.70%
	N2	70	25.70%
M stage			
	M0	228	83.80%
	M1	44	16.20%
TNM stage			
	I	24	8.80%
	II	79	29.00%
	III	128	47.10%
	IV	41	15.10%

Table S5: Primers used in this study	
Gene	Primer Sequence(5’ -3’)
HIF1A-AS2	Forward: TCTGTGGCTCAGTTCCTTTTGT
	Reverse: ATGTAGGAAGTGCCAGAGCC
β-Actin	Forward: ACCGAGCGCGGCTACAG
	Reverse: CTTAATGTCACGCACGATTTCC
ENO2	Forward: AGCCTCTACGGGCATCTATGA
	Reverse: TTCTCAGTCCCATCCAACTCC
PFKFB3	Forward: TTGGCGTCCCCACAAAAGT
	Reverse: AGTTGTAGGAGCTGTACTGCTT

PFKFB4	Forward: GATCCTGAGGTCATAGCTGCCA
	Reverse: CTATCCAGGTCCTCATCTAGCG
PFKL	Forward: CTACGAGGGCTATGAGGGC
	Reverse: GATGACGCACAGGTTGGTGA
GLUT1	Forward: ACCATTGGCTCCGGTATCG
	Reverse: GCTCGCTCCACCACAAACA
GLUT3	Forward: GGAAAGGGCAGGAAGAAGGA
	Reverse: ACAGTCATGAGCGTGGAACAAA
HK2	Forward: ACCCGGGAAAGCAACTGTTTG
	Reverse: TCACCAGGATAAGCCTCACCAG
HK1	Forward: GAGTCTGGACGCGGGAATC
	Reverse: CAGGTGGGCTCCTCATAAGC
LDHA	Forward: GCAGGTGGTTGAGAGTGCTTA
	Reverse: CTTCAAACGGGCCTCTTCCT
ALDOC	Forward: CGCACAGGGAGCTGTCAC
	Reverse: CCTCTGTTTTCCACCCCA
IPO5	Forward: CTGCTGAAGAGGCTAGACAAATG
	Reverse: TCTGCCGCAATATCACAAACTT
HGF	Forward: GACGCAGCTACAAGGGAACA
	Reverse: AGCTCGAAGGCAAAAAGCTG
FOXC1	Forward: GGCGAGCAGAGCTACTACC
	Reverse: TGCGAGTACACGCTCATGG
SP1	Forward: CCATACCCCTTAACCCCG
	Reverse: GAATTTTCACTAATGTTTCCCACC
hsa-miR-217-3p	CATCAGTTCCTAATGCATTGCC
hsa-miR-129-5p	CTTTTTGCGGTCTGGGCTTG
hsa-miR-27a-3p	TTCACAGTGGCTAAGTTCCGC
hsa-miR-33a-5p	GTGCATTGTAGTTGCATTGCA
hsa-miR-199a-3p	ACAGTAGTCTGCACATTGGTTA
hsa-miR-141-3p	TAACACTGTCTGGTAAAGATGG
hsa-miR-153-5p	TCATTTTTGTGATGTTGCAGCT
hsa-miR-4724-5p	AACTGAACCAGGAGTGAGCTTCG
hsa-miR-548n	CAAAAGTAATTGTGGATTTTGT
hsa-miR-6751-3p	ACTGAGCCTCTCTCTCTCCAG
hsa-miR-155-5p	TTAATGCTAATCGTGATAGGGGTT
hsa-miR-200a-3p	TAACACTGTCTGGTAACGATGT
miR-141-3p	Forward: CGTCGCTAACACTGTCTGGTAA
	Reverse: GTGCAGGGTCCGAGGTATTC
U6	Forward: TGCAGACGTGGCAAT
	Reverse: TCAACTGGTGTCGTGG
HIF1A-AS2-promoter	Forward: TTGGGTCACAGTGTTTGTGC
	Reverse: GAGCCACAGATCTCTTAGGTAGC

Table S6:Antibodies used in this study			
Antibody	manufactor	ID	Application
E-cadherin	Abclonal	A20798	WB/IHC
N-cadherin	Abclonal	A19083	WB/IHC
Snail	Abclonal	A5243	WB
β-ACTIN	Proteintech	66009-1-Ig	WB
Goat-anti-Rabbit Secondary Antibody	Proteintech	SA00001-2	WB
Goat-anti-Mouse Secondary Antibody	Proteintech	SA00001-1	WB
Ki67	Servicebio	GB111141	IHC
AGO2	Abcam	ab186733	RIP
FOXC1	CST	8758S	WB
FBP1	CST	59172S	WB
SP1	abcam	ab231778	WB/CHIP
TSG101	abcam	ab125011	WB
HSP70	abcam	ab122844	WB
Annexin A1	abcam	ab214486	WB

**Table S7: Univariate and multivariate analysis of overall survival in 106 CRC patients.**				
	Univariate analysis		Multivariate analysis	
	HR (95%CI)	*P* Value	HR (95%CI)	*P* Value
Age (>=60vs <60)	1.114 (0.578-2.148)	0.747		
Gender (Male vs Female)	1.045 (0.532-2.052)	0.899		
T Stage (T4 vs T1-3)	1.915 (0.999-3.672)	0.05		
N Stage (N1-2 vs N0)	3.950 (1.538-10.10)	0.004*		
M Stage (M1 vs M0)	13.12 (6.254-27.54)	<0.001*		
AJCC stage (Stage III-IV vs Stage I-II)	5.757 (1.767-18.76)	0.004*		
HIF1A-AS2 expression (High vs Low)	3.571 (1.68-7.588)	<0.001*	4.269 (1.785-10.21)	0.001*

HR hazard ratio; CI confidence interval	
* *P*<0.05 indicates that the 95% CI of HR was not including 1	

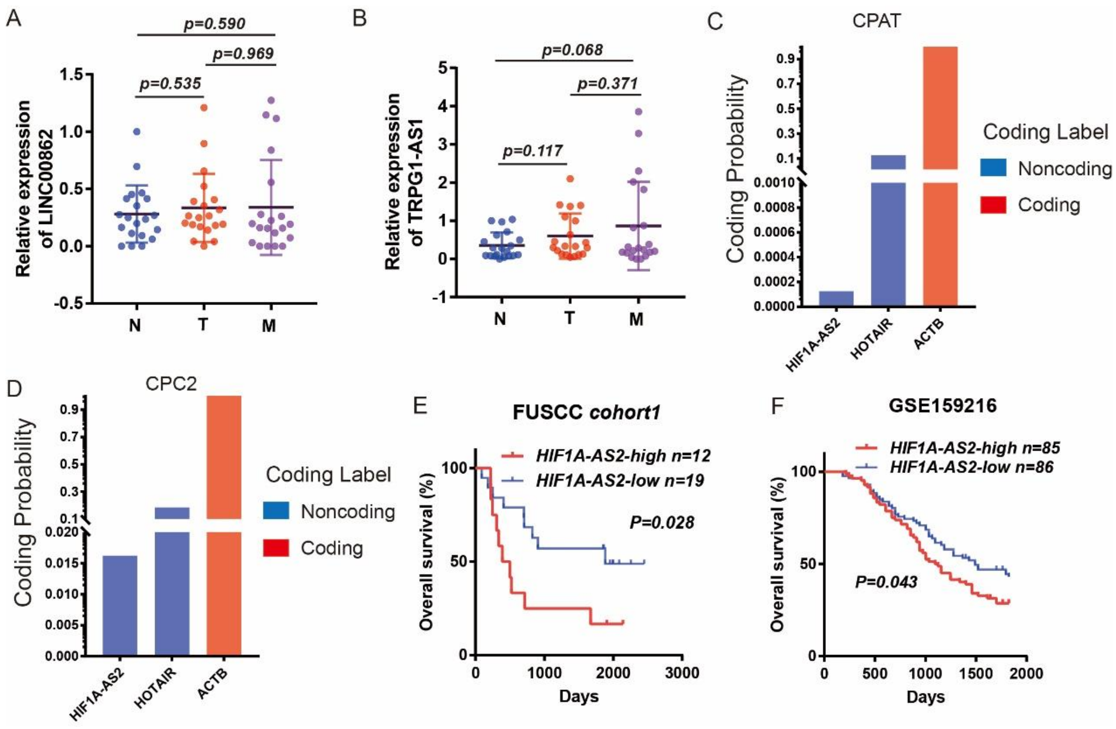

Figure S1 Identification of the lncRNA HIF1A-AS2 in CRC and its clinical significance. **A** Relative LINC00862 expression in normal tissues, primary CRC samples, and CRLM samples from 20 patients. **B** Relative TRPG1-AS1 expression in normal tissues, primary CRC samples, and CRLM samples from 20 patients. **C** The coding potential of HIF1A-AS2 was predicated by the CPAT website. **D** The coding potential of HIF1A-AS2 was predicated by the CPC2 website. **E** Kaplan-Meier curve comparing the OS between HIF1A-AS2-high group and HIF1A-AS2-low group in FUSCC cohort1 with 31 CRLM patients. **F** Kaplan-Meier curve comparing the OS between the HIF1A-AS2-high group and HIF1A-AS2-low group in the GSE159216 cohort.

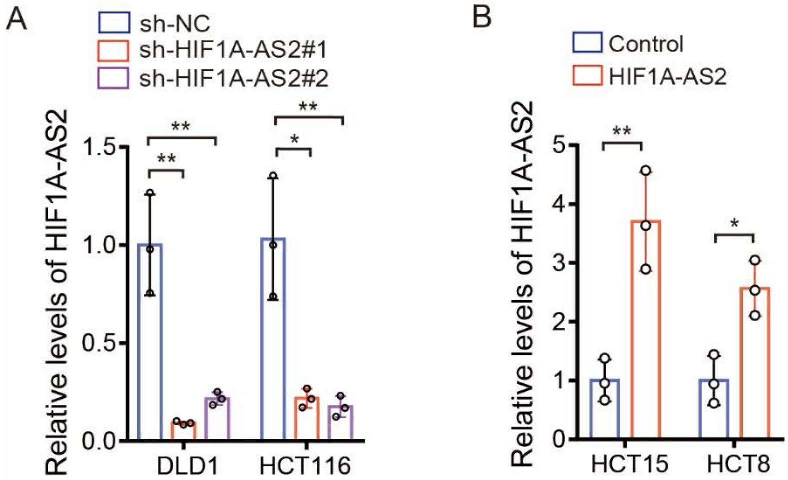

Figure S2 HIF1A-AS2 promotes the malignant phenotype of CRC cells **A-B** RT-qPCR verifying the knockdown effect and overexpression effect of HIF1A-AS2. **P* < 0.05; ***P* < 0.01; ****P* < 0.001; ns, no significance.

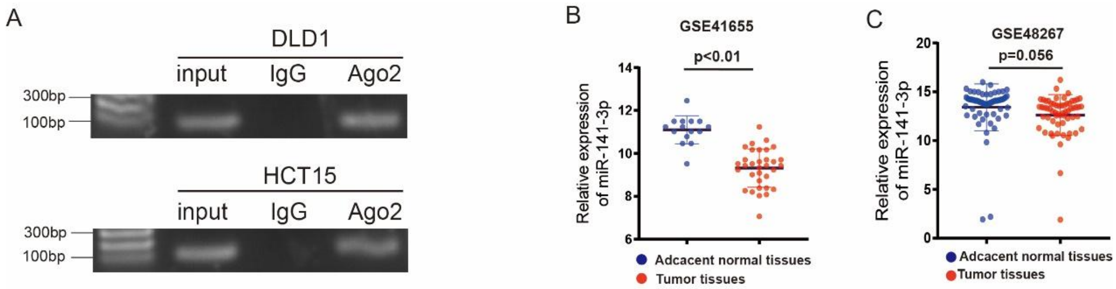

Figure S3 Identification of miR-141-3p as the downstream target of HIF1A-AS2. **A** HIF1A-AS2 level was measured using RNA gel electrophoresis. **B-C** miR-141-3p levels of tumor and normal samples in GSE41655 and GSE48267 datasets.

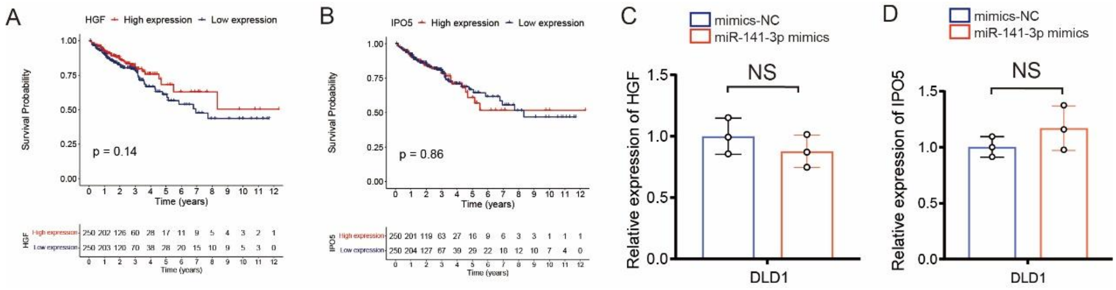

Figure S4 Identification of FOXC1 as the downstream target of miR-141-3p. **A** Kaplan-Meier

curve comparing the OS between the HGF-high group and HGF-low group in the TCGA cohort. **B** Kaplan-Meier curve comparing the OS between IPO5-high group and IPO5-low group in TCGA cohort. **C** Relative HGF expression of CRC cells transfected with miR-141-3p mimics. **D** Relative IPO5 expression of CRC cells transfected with miR-141-3p mimics. **P* < 0.05; ***P* < 0.01; ****P* < 0.001; ns, no significance.

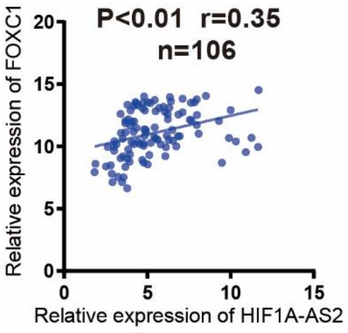

Figure S5 Correlation between HIF1A-AS2 and FOXC1 expression in FUSCC cohort.

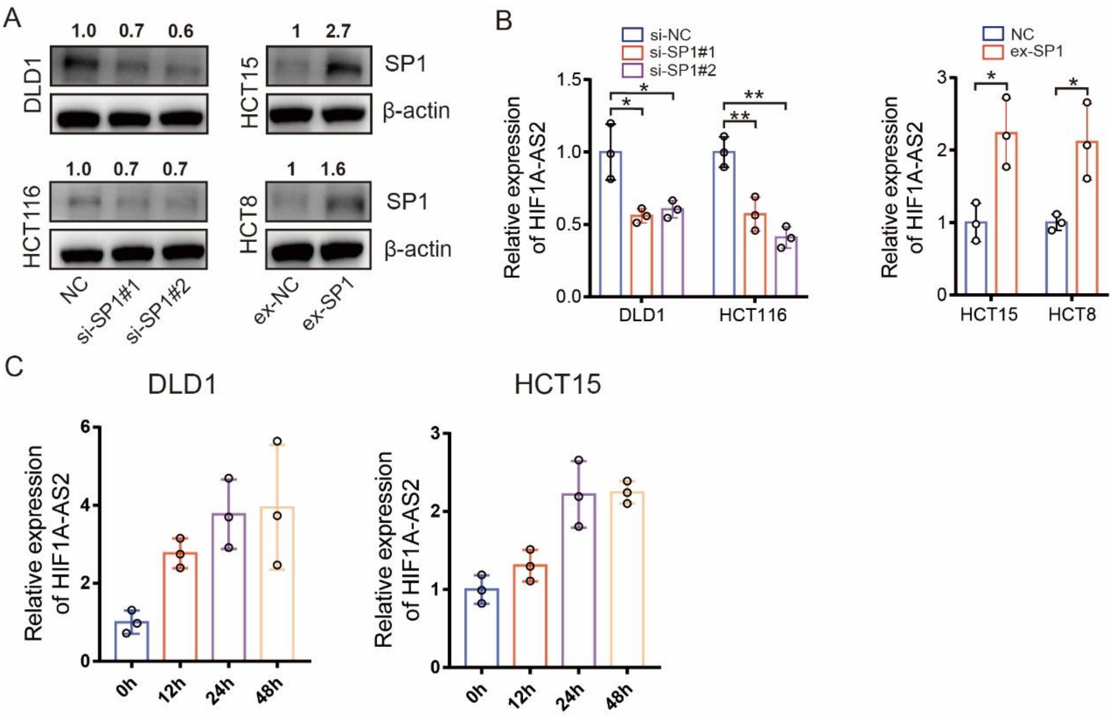

Figure S6 SP1 regulates HIF1A-AS2 expression. **A** The transfection efficiency was verified using Western blot. **B** RT-qPCR showing the expression of HIF1A-AS2 after silencing or

overexpressing SP1. **C** Relative HIF1A-AS2 levels of CRC cells cultured without changing medium for different times. *$P < 0.05$; **$P < 0.01$; ***$P < 0.001$; ns, no significance.

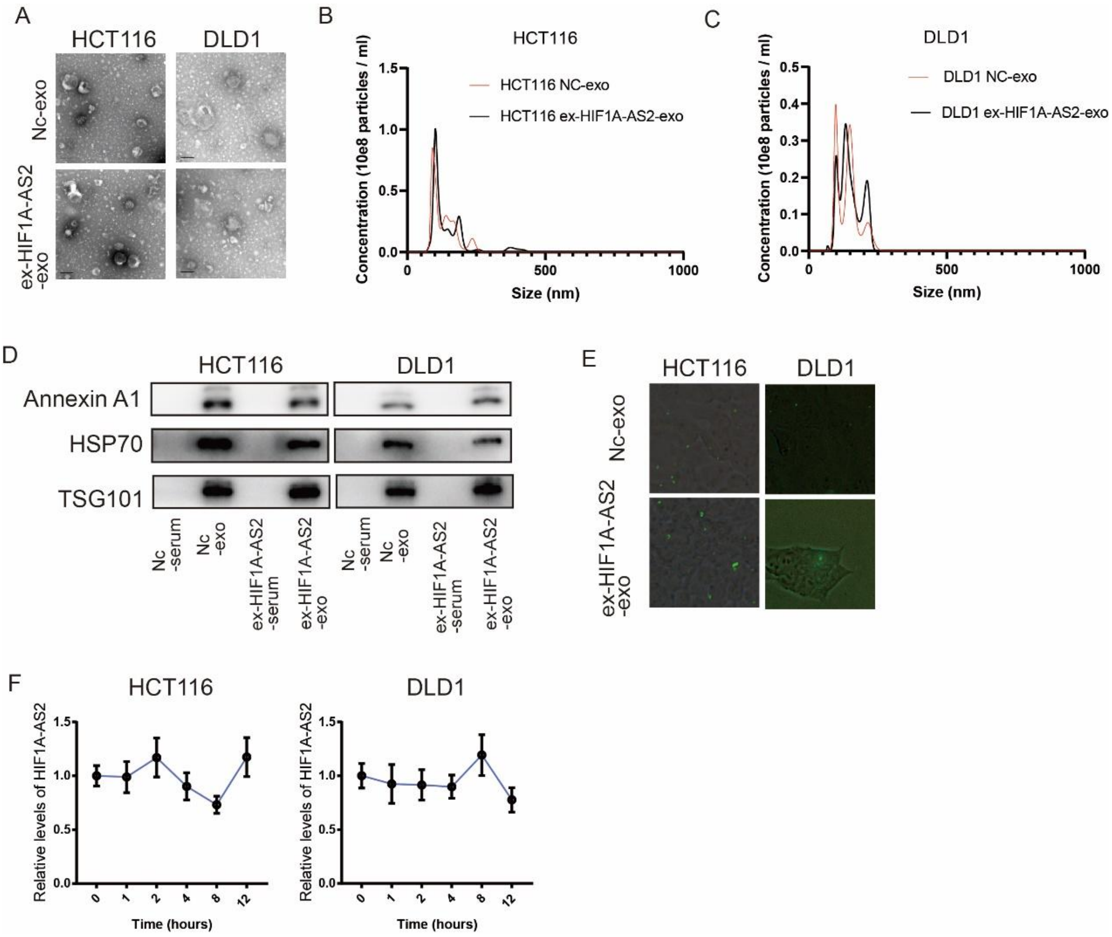

Figure S7 Exosomal HIF1A-AS2 promotes CRC progression *in vivo and in vitro*. **A** Representative transmission electron microscopy (TEM) images of exosomes from CRC cells. Scale bar = 100 nm. **B** Histogram showing the size of exosomes derived from HCT116 cells. **C** Histogram showing the size of exosomes derived from DLD1 cells. **D** Western blot showing the expression of exosomal markers in each sample. **E** Representative images of stained exosomes that were taken up by CRC cells. **F** Relative level of HIF1A-AS2 in exosomes that were stored at room temperature for different hours. *$P < 0.05$; **$P < 0.01$; ***$P < 0.001$; ns, no significance.
