# Supplementary figures and images for: HIF1A-AS2 promotes the metabolic reprogramming and progression of colorectal cancer via miR-141-3p/FOXC1 axis
Source: Cell Death Dis. 2024 Sep 3;15(9):645. doi: 10.1038/s41419-024-06958-2 (PMC11372083; doi:10.1038/s41419-024-06958-2)

Figure2I

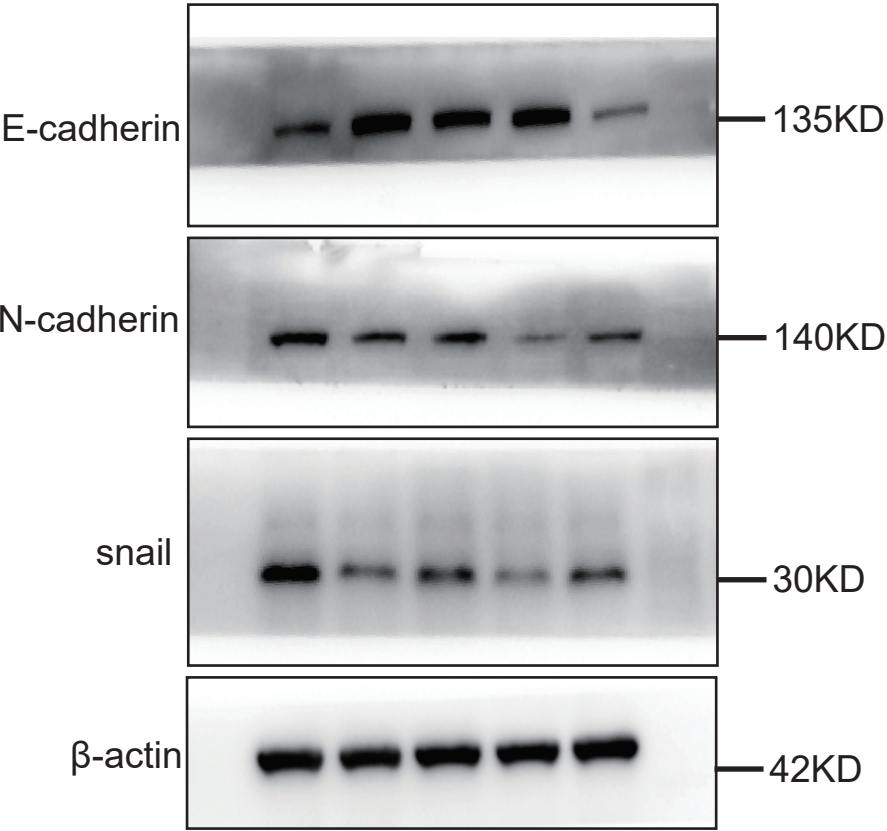

Figure6E

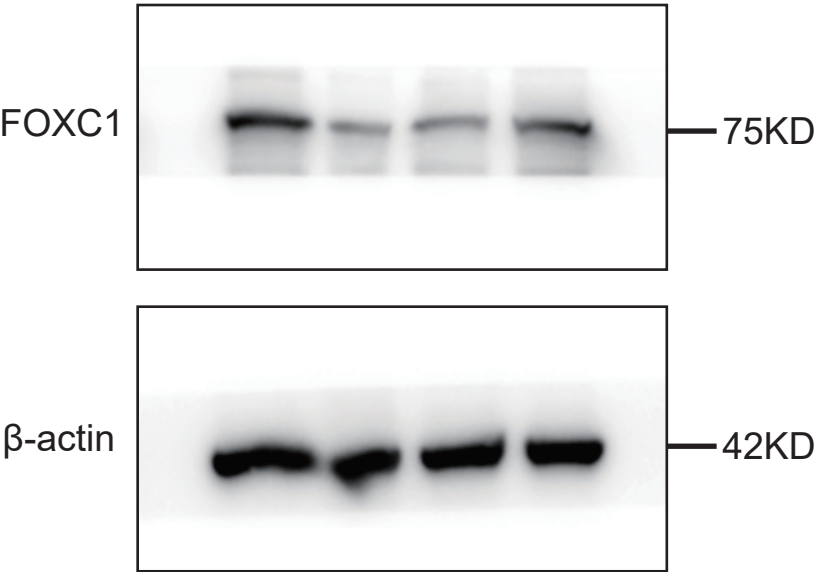

Figure7A

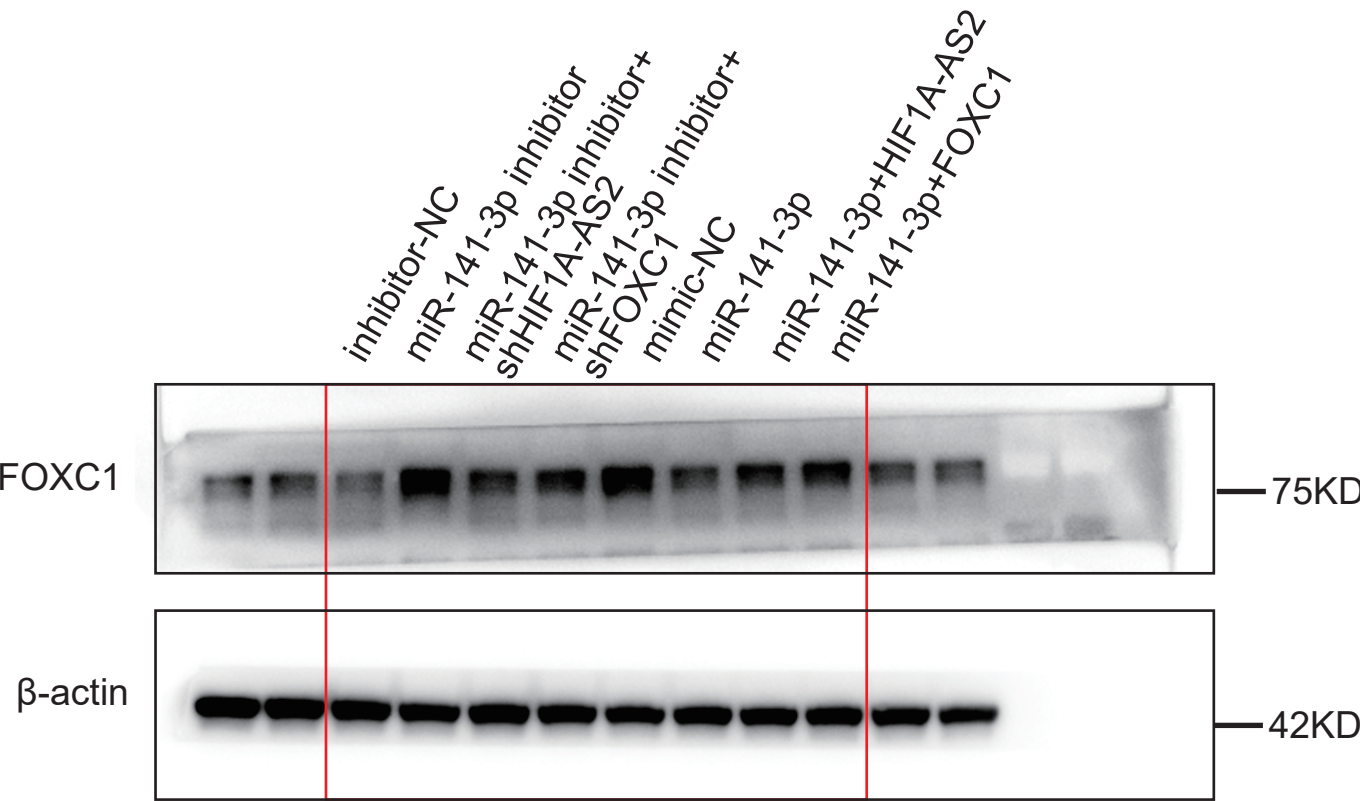

Figure7M

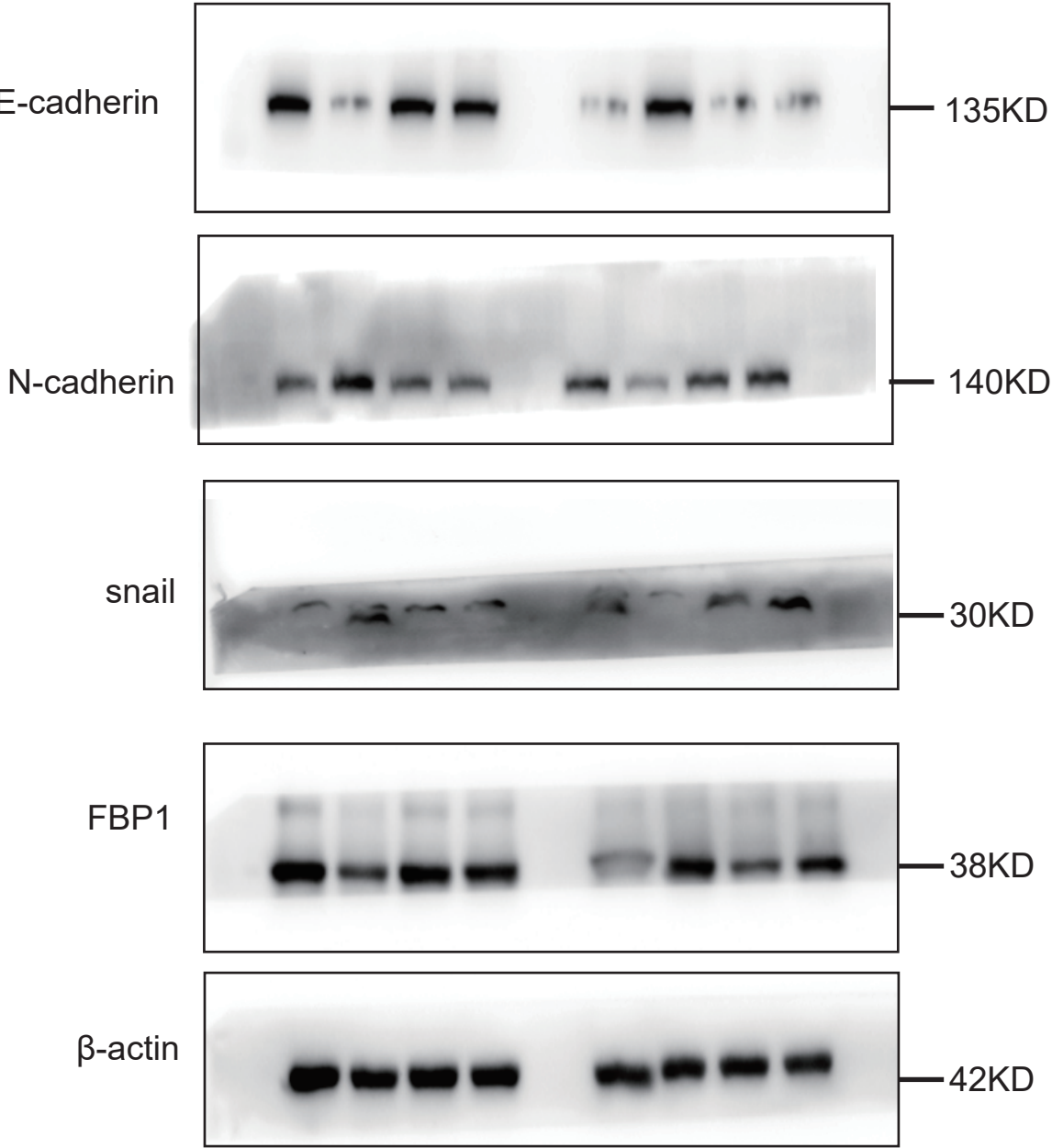

FigureS6A

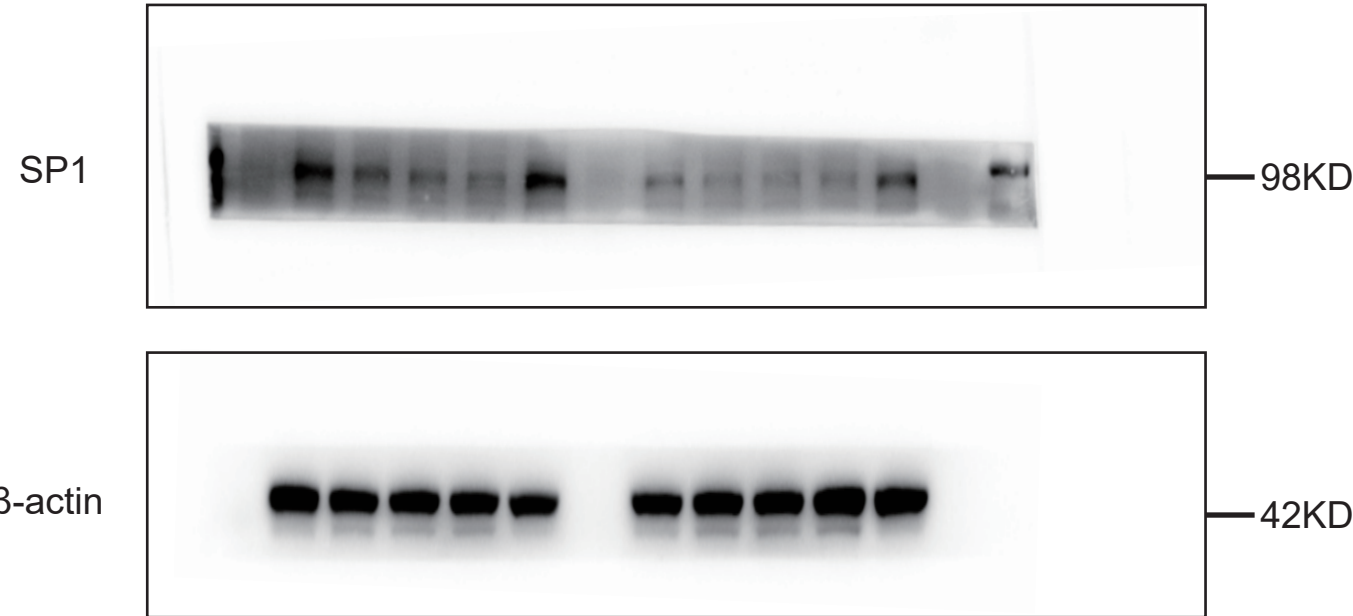

FIGURE8Q

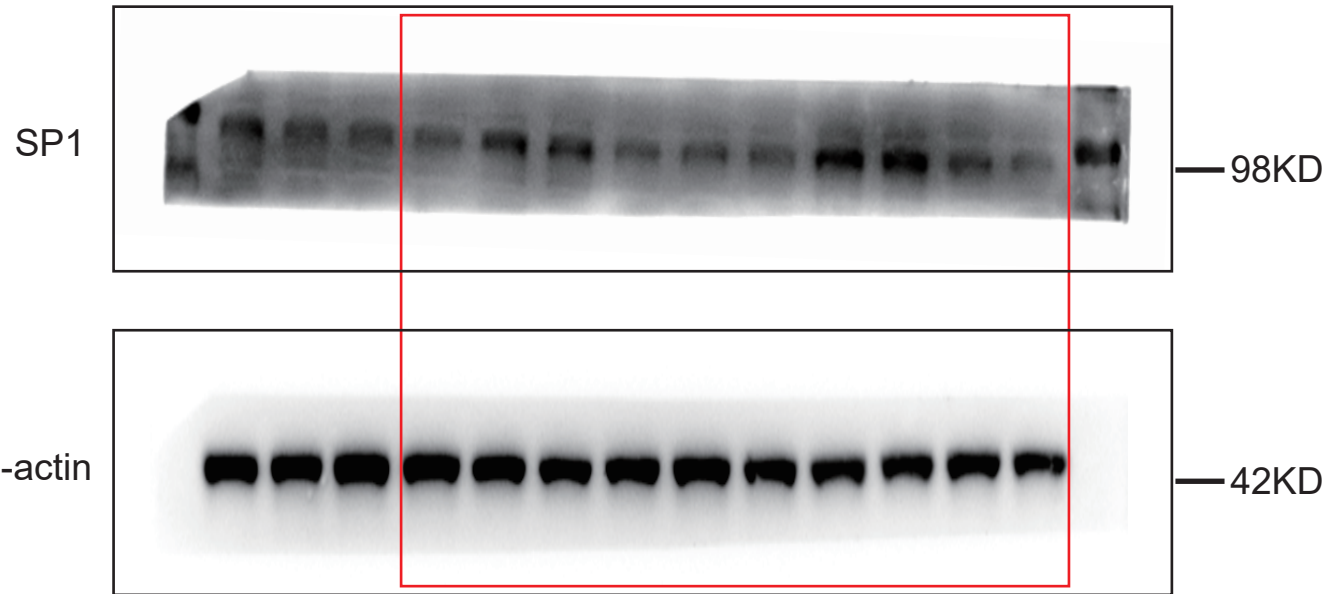

FIGURES7D

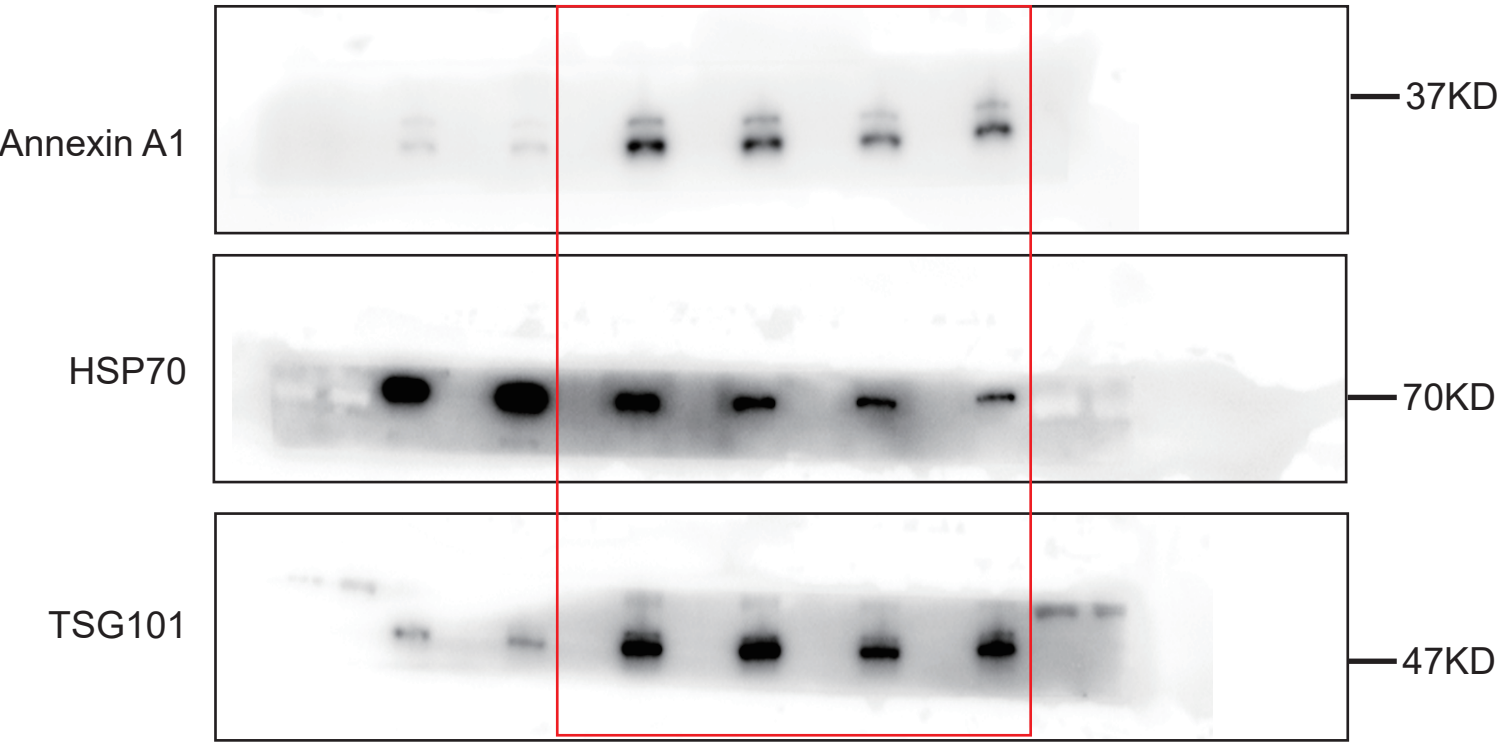

Supplement: Supplementary file 2 — original data [file 41419_2024_6958_MOESM2_ESM.pdf]
